# Human Pluripotent Stem Cell Fate Regulation by SMARCB1

**DOI:** 10.1016/j.stemcr.2020.10.002

**Published:** 2020-10-29

**Authors:** Ilana Carmel-Gross, Etgar Levy, Leah Armon, Orly Yaron, Hiba Waldman Ben-Asher, Achia Urbach

**Affiliations:** 1 The Mina and Everard Goodman Faculty of Life Sciences, Bar-Ilan University, Ramat Gan 5290002, Israel

**Keywords:** pluripotent stem cell, SMARCB1, SWI/SNF complex, extra cellular matrix

## Abstract

Epigenetic regulation by the SWI/SNF complex is essential for normal self-renewal capacity and pluripotency of human pluripotent stem cells (hPSCs). It has been shown that different subunits of the complex have a distinct role in this regulation. Specifically, the SMARCB1 subunit has been shown to regulate the activity of enhancers in diverse types of cells, including hPSCs. Here, we report the establishment of conditional hPSC lines, enabling control of SMARCB1 expression from complete loss of function to significant overexpression. Using this system, we show that any deviation from normal SMARCB1 expression leads to cell differentiation. We further found that SMARCB1 expression is not required for differentiation of hPSCs into progenitor cells, but rather for later stages of differentiation. Finally, we identify SMARCB1 as a critical player in regulation of cell-cell and cell-ECM interactions in hPSCs and show that this regulation is mediated at least in part by the WNT pathway.

## Highlights

• SMARCB1 levels have to be precisely regulated in hPSCs
• SMARCB1 loss of function affects hPSC self-renewal capacity and pluripotency
• SMARCB1 overexpression leads to hPSC differentiation
• SMARCB1 is required for normal cell-cell and cell-ECM interactions in hPSCs

## Teaser

Using conditional loss of function and overexpression systems, Urbach and colleagues show that any deviation from SMARCB1 normal expression levels affects the self-renewal capacity of the hPSCs. They further show that SMARCB1 is required to maintain the differentiation capacity of the cells. Finally, they demonstrate the critical role of SMARCB1 in cell-cell and cell-ECM interactions in hPSCs.

## Introduction

The SWI/SNF chromatin remodeling complex controls the chromatin structure by nucleosome mobilization. The human SWI/SNF complex contains a single ATPase (SMARCA2 also known as BRM or SMARCA4 also known as BRG1), 3 core subunits, and 7 to 15 additional accessory subunits. The specific composition of the subunits varies between different tissues (for review, see Masliah-Planchon et al., 2014). The chromatin remodeling capacity of the complex enables tight regulation of gene expression. Therefore, it plays an important role in regulation of many cellular processes (Masliah-Planchon et al., 2014), including during preimplantation embryonic development (Kim et al., 2001; Klochendler-Yeivin et al., 2000). In agreement with its role during early embryonic development, it has been shown that SMARCA4 (Kidder et al., 2009), the core subunits ARID1A/B (BAF250a/b) (Gao et al., 2008; Yan et al., 2008), SMARCB1 (Schaniel et al., 2009), and the specific composition of the accessory subunits (Ho et al., 2009) are essential to maintain the self-renewal capacity and pluripotency of mouse embryonic stem cells (mESCs). These observations raise the question of whether SMARCB1 plays a similar role also in human pluripotent stem cells (hPSCs), which are fundamentally different from naive mESCs (Nichols and Smith, 2009; Yilmaz and Benvenisty, 2019). To address this question, Zhang et al. (2014) compared the role of several SWI/SNF subunits between mESCs and hPSCs. This analysis revealed several significant differences between the composition and function of the various SWI/SNF subunits in mouse versus human hPSCs.

The SWI/SNF core subunit SMARCB1 (also known as INI1, SNF5, and BAF47) has been shown to control recruitment of the SWI/SNF complex to enhancers and bivalent promoters, thus regulating their activation (Alver et al., 2017; Nakayama et al., 2017; Wang et al., 2017). The fact that SMARCB1 loss of function (LOF) is the sole mutation found in the vast majority of rhabdoid tumors (highly aggressive pediatric tumors arising mainly in the brain (AT/RT) and kidneys (for review see [Lee et al., 2012; Masliah-Planchon et al., 2014]) emphasizes its critical role in cell fate epigenetic regulation.

Recently, Langer et al. (2019) have found that SMARCB1 suppresses the activity of hPSC super-enhancers during neuronal differentiation and thus enables the pluripotent cells to differentiate toward this lineage. This study provides a novel and important insight into the role of SMARCB1 in hPSCs. Yet, it is based on partial downregulation of the gene (∼80% reduction compared with normal hPSCs). Therefore, a complementary system of SMARCB1 complete LOF is required to fully understand the involvement of SMARCB1 in hPSC fate regulation.

Here, we report the establishment of human embryonic stem cell (hESC) and human induced pluripotent stem cell (hiPSC) lines enabling the control of SMARCB1 expression levels ranging from null expression to a significant overexpression. We found that both SMARCB1 LOF and SMARCB1 overexpression impair the self-renewal capacity of the cells. We further show that SMARCB1 complete LOF affects the differentiation capacity of hPSCs; however, in a different manner than partial SMARCB1 LOF. Finally, our data provide the first indication for the role of SMARCB1 in the maintenance of normal cell-cell and cell-extracellular matrix (ECM) interactions in hPSCs and reveal the involvement of the WNT pathway in these processes.

## Results

### Establishment of Conditional SMARCB1 LOF hPSC Lines

**Figure 1:**
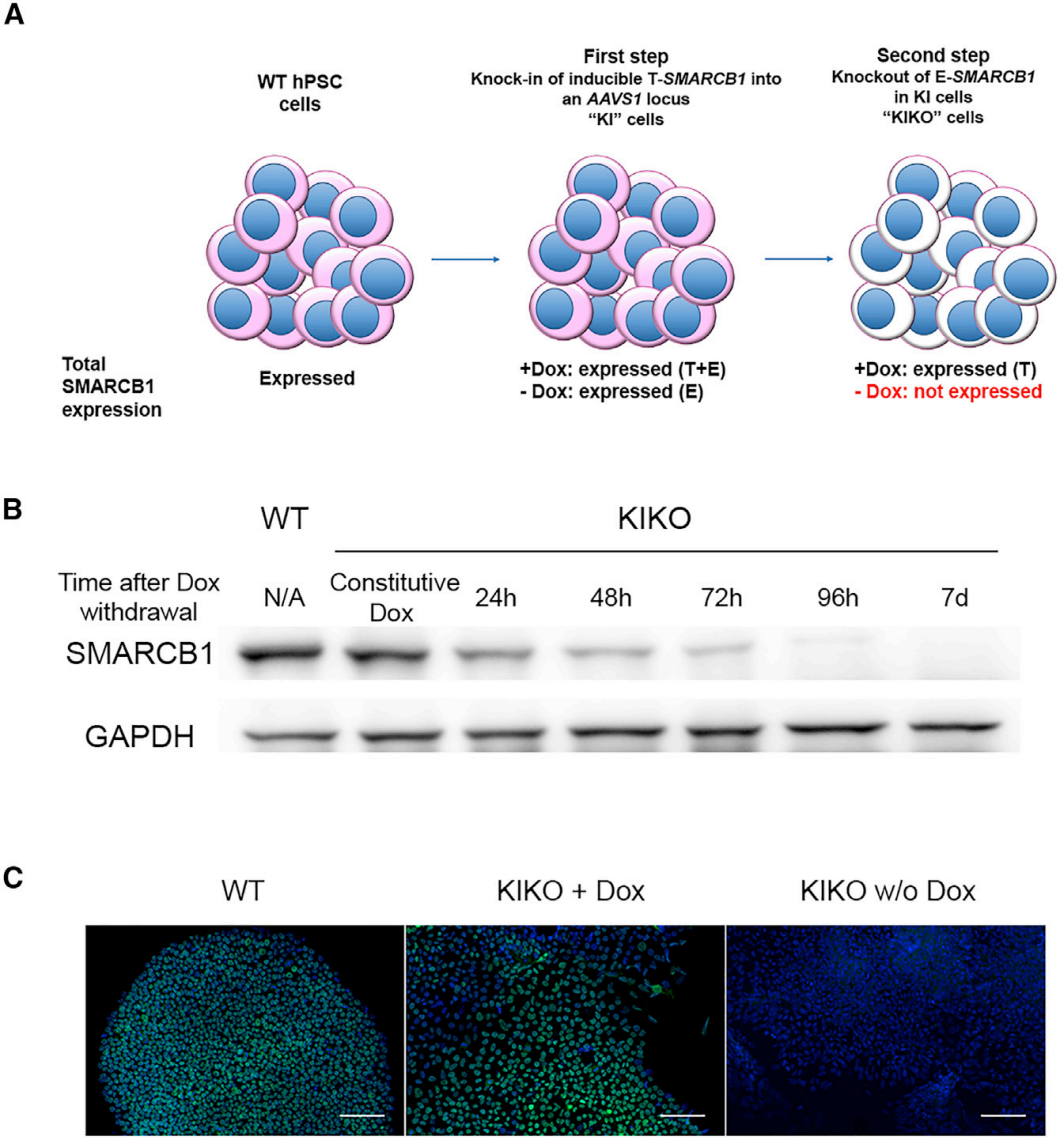
Conditional SMARCB1 LOF in hPSCs (A) Scheme describing the two-step approach for SMARCB1 conditional knockout in hPSCs. In the presence of Dox, the KI cells express both the endogenous (E) and the transgenic (T) SMARCB1, while the KIKO cells express only the transgenic SMARCB1. In the absence of Dox, there is no expression of SMARCB1 in the KIKO cells. (B) Western blot analysis of hESC wild-type (WT) and KIKO cells in the presence of Dox and at different time points after Dox withdrawal. (C) Immunostaining of single-cell-derived KIKO clones from hESCs in the presence of Dox or 96 h after Dox withdrawal. Green, SMARCB1; blue, DAPI. Scale bar, 100 μm.

To study the role of SMARCB1 in hPSCs, we first aimed to target *SMARCB1* using the CRISPR-Cas9 system with two gRNAs directed upstream and downstream of *SMARCB1* exon 2 (Figure S1A). We first verified this system at the population level and found, as expected, that the transfected cells show patches of SMARCB1-negative cells (Figure S1B). Next, we screened by PCR single-cell-derived clones and found that 23% (15/64) of the clones were heterozygous for exon 2 deletion (*SMARCB1*^+/−^). Notably, however, we did not find even a single *SMARCB1*^−/−^ clone. These results suggest that SMARCB1 LOF might have a negative effect on the growth/morphology of hPSCs. Therefore, we established a conditional SMARCB1 LOF system based on a two-step approach. First, we knocked in a *SMARCB1* conditional (Tet-On) overexpression cassette into the AAVS1 locus of hPSCs (herein KI cells). Next, we targeted the endogenous *SMARCB1* in the KI cells in the presence of low doxycycline (Dox) concentration (12 ng/mL) to maintain normal SMARCB1 levels. This strategy enabled us to isolate clones of hESCs and of hiPSCs with a homozygous deletion of exon 2 (Figure S1C) in reasonable efficiency (3/58 and 4/17 homozygous clones in hESC and hiPSC lines, respectively). These clones (herein KIKO cells, knockout on the background of knockin) retain normal levels of SMARCB1 expression in the presence of Dox (herein KIKO + Dox) but completely fail to express the gene within 4 days upon Dox withdrawal (herein KIKO w/o Dox) (Figures 1A–1C and S1D).

### Deviations from Normal SMARCB1 Levels Affect the Self-Renewal Capacity of hPSCs

**Figure 2:**
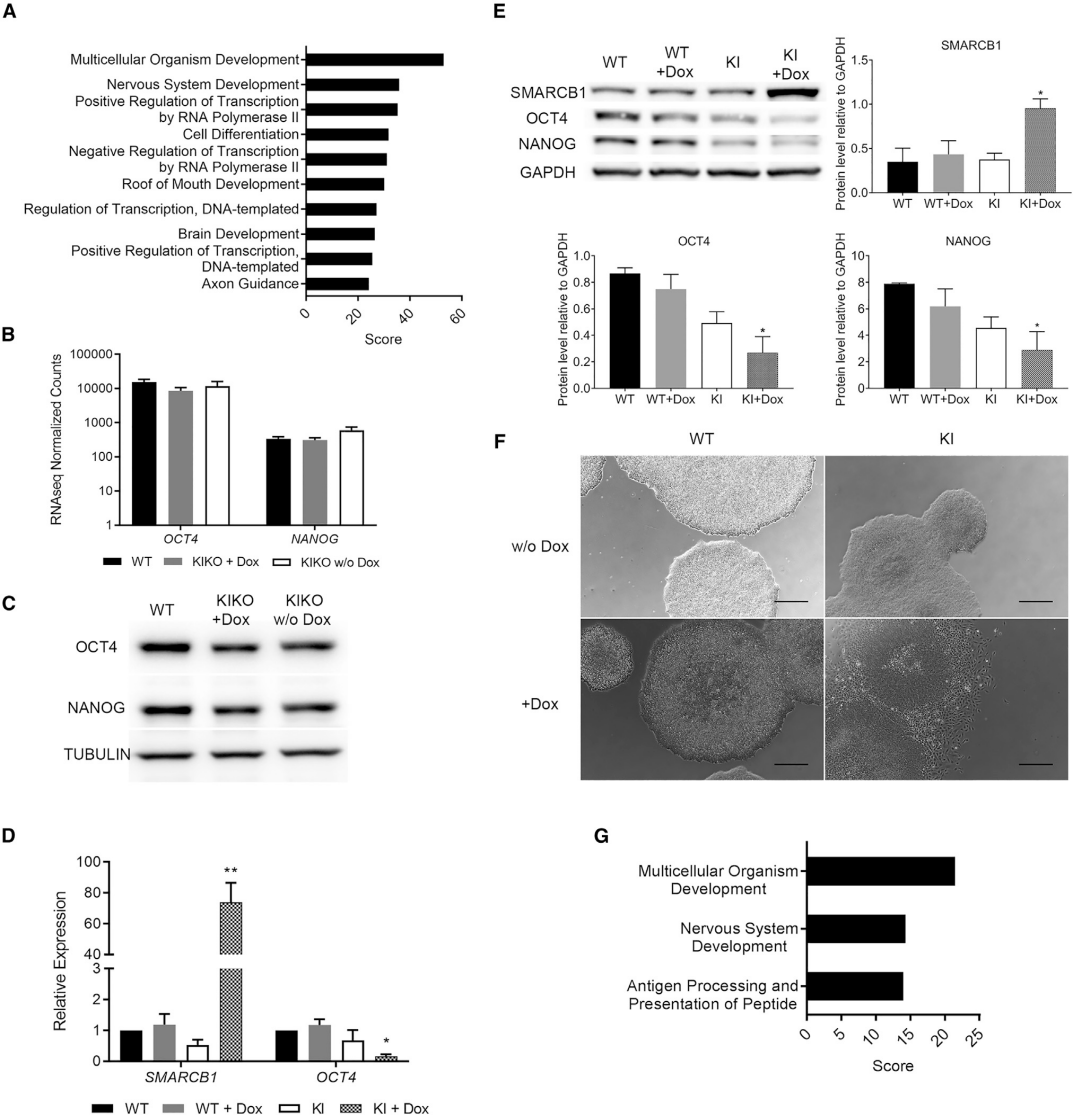
The Effect of SMARCB1 Misregulation on Self-Renewal Capacity of hPSCs (A) Top high scored gene ontology (GO) terms of genes upregulated upon SMARCB1 complete LOF. (B and C) OCT4 and NANOG levels in controls and SMARCB1 LOF cells as determined by RNA-seq (B) and western blot analysis (C). (D) qRT-PCR analysis for *SMARCB1* and *OCT4* in controls and SMARCB1 overexpressing cells. (E) Representative image and quantification of western blot analysis for SMARCB1, OCT4, and NANOG levels upon SMARCB1 overexpression. (F) Phase-contrast images of representative hESC colonies. Scale bar, 500 μm. (G) High scored GO terms of genes upregulated upon SMARCB1 overexpression. All quantitative data are represented as means of three biological replicates. qRT-PCR data are normalized to WT. ^∗^p < 0.05, ^∗∗^p < 0.001. Statistical analysis was performed using multiple t tests with false discovery rate (FDR) adjustment (D) and one-way ANOVA with Dunnett's post hoc test (E).

Previous studies revealed that downregulation of SMARCB1 (Langer et al., 2019) or other SWI/SNF core subunits (Zhang et al., 2014) leads to rapid upregulation of differentiation markers, while pluripotent genes, such as *OCT4* and *NANOG* are still normally expressed. To examine if SMARCB1 complete LOF has the same effect on hPSCs, we performed RNA sequencing (RNA-seq) of KIKO and control cells 7 days after Dox withdrawal (3 days after complete SMARCB1 LOF). Our RNA-seq analysis revealed ∼240 upregulated and ∼440 downregulated genes upon SMARCB1 LOF. Gene ontology (GO) annotation of the RNA-seq data indicates a rapid upregulation of biological processes related to multicellular organism development, and specifically neuronal development (Figure 2A). As in the abovementioned studies, these changes in gene expression were not accompanied by downregulation of pluripotent markers, such as OCT4 and NANOG (Figures 2B and 2C).

Next, we studied whether SMARCB1 overexpression also affects the self-renewal capacity of the cells. For this purpose, we cultured the KI cells for 14 days in the presence of high Dox concentration (50 ng/μL), which led to a significant increase in SMARCB1 levels (Figures 2D and 2E). This upregulation led to cell differentiation, as demonstrated by the significant downregulation of OCT4 and NANOG (Figures 2D and 2E), morphological changes (Figure 2F), and GO annotation of the upregulated genes found by RNA-seq (Figure 2G). Notably, the downregulation of the pluripotency markers appeared already after a short time (7 days) of Dox treatment (data not shown). Overall, these results, together with the previous observations (Langer et al., 2019) regarding the effect of partial SMARCB1 downregulation, indicate that SMARCB1 has to be precisely regulated in hPSCs in order to maintain their self-renewal capacity.

### SMARCB1 Complete LOF Affects the Differentiation Capacity of hPSCs

**Figure 3:**
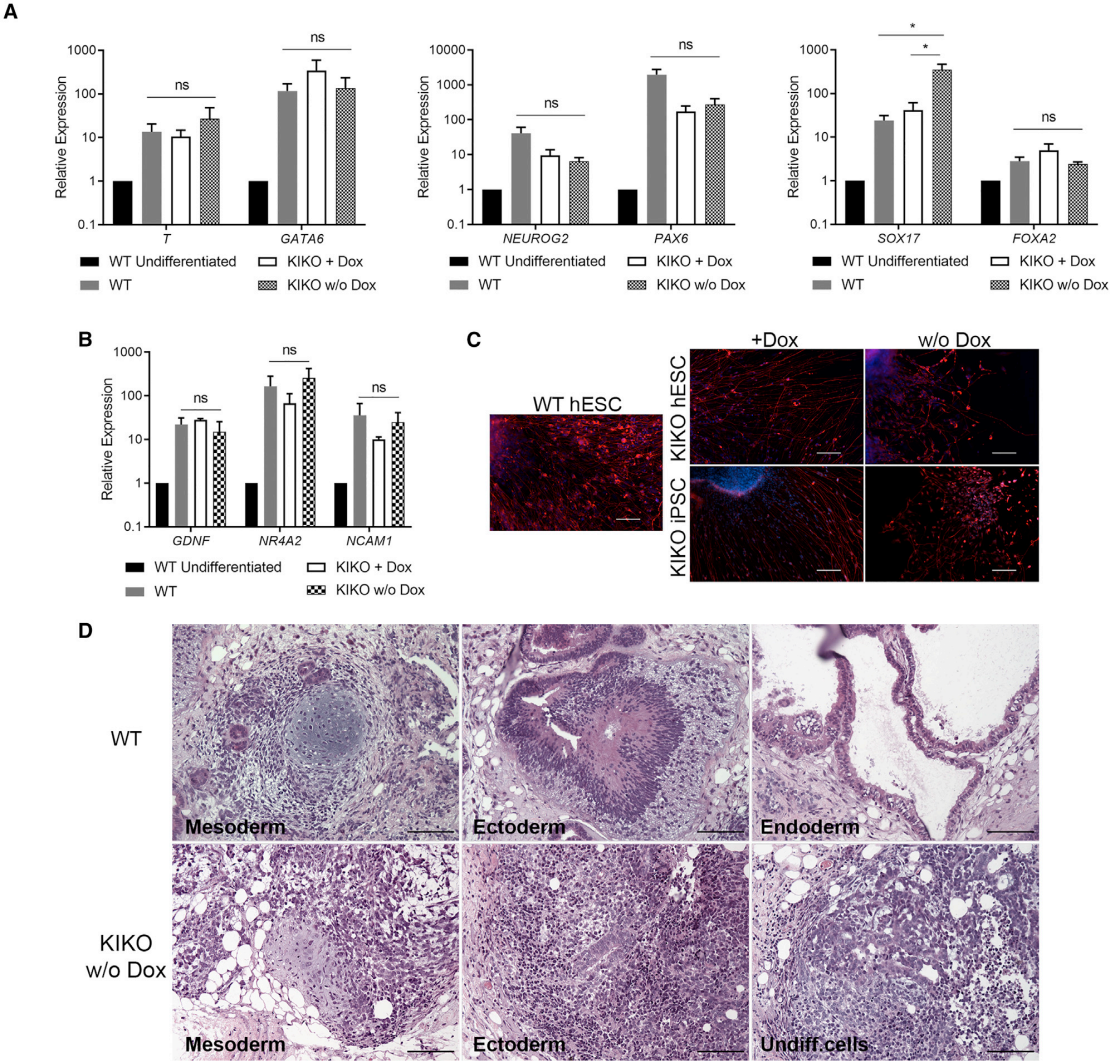
The Effect of SMARCB1 LOF on hPSC Differentiation (A) qRT-PCR analysis of mesodermal (left), ectodermal (middle), and endodermal (right) genes upon direct differentiation. (B) qRT-PCR analysis of neuronal markers upon direct neuronal differentiation. qRT-PCR data in (A and B) are represented as mean of three biological replicates ± SEM. Data are normalized to WT undifferentiated cells. Statistical analysis using one-way ANOVA with FDR correction. ns, not significant. ^∗^p < 0.05. (C) Immunofluorescent staining of neurons derived from control and SMARCB1 LOF cells. Red, NF-H; blue, DAPI. Scale bar, 100 μm. (D) H&E staining of teratomas derived from WT and SMARCB1 LOF hPSCs. Scale bar, 50 μm.

To study the effect of SMARCB1 complete LOF on the pluripotency of the cells, we first evaluated the capacity of the cells to differentiate *in vitro* into the three germ layers using a direct differentiation method. We found that the SMARCB1 LOF cells retained their capacity to differentiate into the mesoderm, ectoderm, and endoderm lineages similar to the control cells (Figure 3A). These results indicate that SMARCB1 expression is not required for differentiation of the hPSCs into cells of the three germ layers. Notably, the expression of *PAX6* and *NEUROG2* upon ectodermal differentiation (Figure 3A) suggests that, by contrast to partial SMARCB1 LOF (Langer et al., 2019), a complete silencing of the gene does not abrogate hPSC neuronal differentiation capacity. To further confirm this observation, we used a direct neuronal differentiation protocol (Birenboim et al., 2013). Indeed, a gene expression analysis revealed that SMARCB1 LOF cells successfully differentiated into the neuronal lineage (Figure 3B). Interestingly, however, the neuronal differentiation seems to be impaired in these cells (Figure 3C). Overall, these results suggest that, while SMARCB1 is not essential for the differentiation of the cells into progenitor cells of the three germ layers, its expression is required for the subsequent differentiation at least into the neuronal lineage. To validate this assumption, we performed a teratoma formation assay. The teratomas derived from the SMARCB1 LOF cells contained mesodermal derivatives, such as bone and cartilage (Figure 3D), but mature ectodermal and endodermal cells were scarcely detected in these teratomas except for immature neural tube structures (Figure 3D, bottom middle). Moreover, patches of undifferentiated cells were found across these teratomas (Figure 3D, bottom middle and right). The fact that these patches were OCT4 negative (data not shown) indicates that they comprise cells that started to differentiate, but failed to complete their differentiation. These *in vivo* results strongly support the assumption that SMARCB1 is required for maturation of the ectodermal lineage (and probably also for endodermal derivatives).

### SMARCB1 Is Required for Cell-ECM and Cell-Cell Interactions of hPSCs

**Figure 4:**
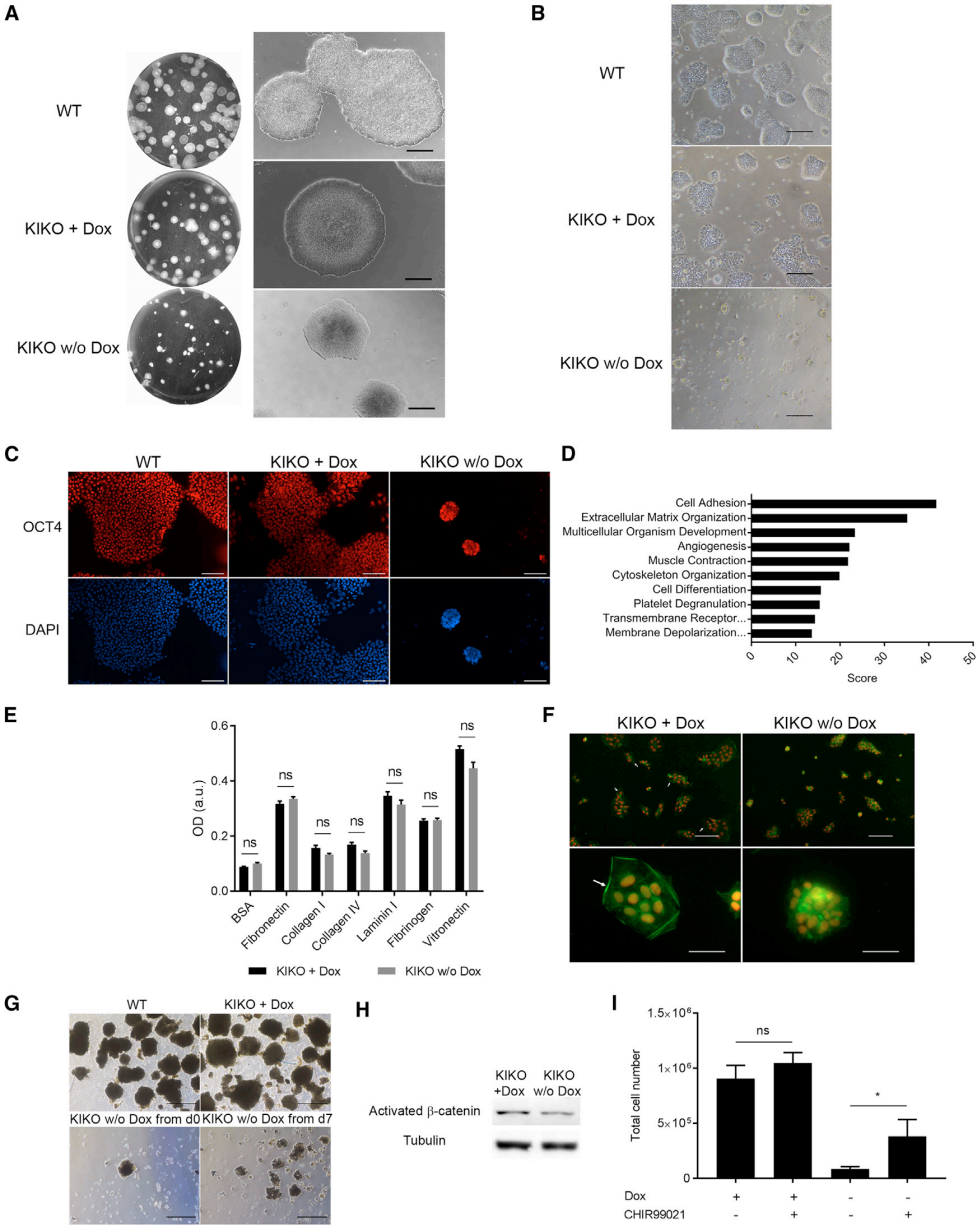
Abnormal Cell-Cell and Cell-ECM Interactions in SMARCB1 Complete LOF hPSCs (A) Controls and SMARCB1 LOF hPSC colonies. Left: low-magnification, whole-plate imaging. Right: higher magnification of representative colonies. Scale bar, 500 μm. (B) Controls and complete SMARCB1 LOF colonies 72 h after single-cell plating. Scale bar, 500 μm. (C) Immunostaining for OCT4, 72 h after single-cell plating. Scale bar, 100 μm. (D) Top 10 high scored GO terms of genes downregulated upon SMARCB1 complete LOF. (E) Results of ECM adhesion assay for SMARCB1 LOF and control cells. The assay was done on different types of ECM proteins, as indicated in the figure. BSA was used as a negative control. n = 4 for each condition. (F) Representative images of Phalloidin (green) and OCT4 (red) immunostaining. Upper panels: low magnification. Scale bar, 100 μm. Bottom panels: high magnification. Scale bar, 50 μm. The actin fence organization of the actin fibers appears at the edges of the control colonies (white arrows), but not in the SMARC1 LOF cells. (G) EBs derived from control and KIKO cells upon Dox withdrawal at two different time points. Scale bar, 500 μm. (H) A representative image of western blot for activated β-catenin (tubulin shown as a loading control). (I) Cell number quantification of SMARCB1 LOF and control colonies grown attached to the plate upon single-cell passaging in the presence or absence of CHIR99021 treatment. n = 3; ns, not significant, ^∗^p < 0.05. Statistical analysis was performed using one-way ANOVA with FDR correction.

In addition to the abovementioned phenotypes, the most prominent effect of SMARCB1 LOF was significant morphological changes of the hPSC colonies. Three days after complete SMARCB1 silencing, the cells started to grow as multilayered 3D colonies instead of spreading horizontally as monolayer colonies (Figures 4A and S2A). These morphological alterations were not accompanied by significant changes in the proliferation (Figures S2B and S2C) and apoptosis of the cells (Figure S2D). Moreover, a single-cell passaging of the SMARCB1 LOF cells significantly abrogated their capacity to generate typical hPSC colonies. Specifically, the single-cell-derived colonies were small and compacted, and many of them failed to stay attached to the plate (Figure 4B), but retained the expression of pluripotency markers (Figures 4C and S2E). Altogether, these observations suggest that SMARCB1 LOF affects the capacity of hPSCs to establish normal interactions with the ECM. Indeed, RNA-seq analysis revealed that pathways and biological processes related to cell adhesion and ECM organization are significantly downregulated in the absence of SMARCB1 expression (Figure 4D). This effect of SMARCB1 LOF is not due to inability of the mutated cells to adhere to the ECM as evident by cell adhesion assay (Figure 4E). Rather, it is probably the result of a failure to maintain the interactions between the cells and the ECM. A unique organization of actin fibers into ventral stress fibers called actin fence is known to regulate hPSC-ECM interactions (Närvä et al., 2017; Stubb et al., 2019). To explore whether this organization is affected by SMARCB1 LOF, we stained the cells with phalloidin. Remarkably, the SMARCB1 LOF cells failed to establish this unique actin fence organization (Figure 4F). Overall, these observations demonstrate an important role of SMARCB1 in the regulation of the interaction between hPSCs and the ECM.

To explore if SMARCB1 is required also for cell-cell interaction in hPSCs, we studied their aggregation capacity by embryonic bodies (EBs) formation assay. By contrast to the control cells, the SMARCB1 LOF cells (Dox withdrawal at day 0 of the assay) almost completely failed to generate EBs. Moreover, SMARCB1 LOF affected the cell ability to generate EBs even when it was induced at later stage of the assay (Dox withdrawal at day 7 of the assay) (Figures 4G and S2F). These results indicate that SMARCB1 expression is also required for cell-cell interactions in hPSCs.

Finally, our RNA-seq analysis revealed downregulation of several genes related to the WNT signaling pathway (GO:0016055, *Z* score 13.28). In accordance, we found that the SMARCB1 LOF leads to a significant reduction in activated β-catenin levels (Figure 4H). These results suggest that the effect of SMARCB1 LOF on hPSC fate is mediated at least in part via inhibition of the WNT pathway. To validate this hypothesis, we explored the effect of the WNT pathway activation by CHIR99021 treatment (2 μM) on SMARCB1 LOF cells. Indeed, this treatment significantly increased the number of SMARCB1 LOF colonies grown attached to the plate upon single-cell passaging (Figure 4I) and thus confirm the interplay between SMARCB1 and WNT pathways in hPSCs.

## Discussion

The self-renewal and the differentiation capacity of pluripotent stem cells are tightly epigenetically regulated (Bibikova et al., 2008; Meshorer and Misteli, 2006; Yilmaz and Benvenisty, 2019). Therefore, it is expected that normal expression and function of the SWI/SNF complex will be essential for the maintenance of hPSCs. Here, we show that complete SMARCB1 LOF, as well as SMARCB1 overexpression, leads to hPSC differentiation. These results are in agreement with those of Langer et al. (2019) showing that partial SMARCB1 LOF in hPSCs also leads to hPSC differentiation. A similar effect on hPSC self-renewal capacity was observed upon downregulation of SMARCA4, the SWI/SNF catalytic subunit (Zhang et al., 2014). Yet, this effect is not common for all SWI/SNF core subunits. For example, SMARCC1 depletion has no overt effect on the cells (Zhang et al., 2014).

While the abovementioned results reveal a similar role for SMARCA4 and SMARCB1 in hPSC self-renewal regulation, it appears that these two subunits play a different role in the regulation of hPSC pluripotency. SMARCA4-deficient hPSCs show a reduced capacity to differentiate into the mesodermal lineage but retain their ectodermal differentiation capacity, including neuronal differentiation (Zhang et al., 2014). On the contrary, our results show that SMARCB1 expression is not required for mesodermal differentiation of hPSCs, but rather for neuronal differentiation. Interestingly and counterintuitively, the effect of partial SMARCB1 LOF on neuronal differentiation appears to be more severe than the effect observed upon complete SMARCB1 LOF. Specifically, Langer et al. showed that SMARCB1 functions by silencing of hPSC-specific super-enhancers during the differentiation into the neuronal lineage, and thus SMARCB1 downregulation impairs neuronal induction from hPSCs. In contrast, we found that complete SMARCB1 LOF hPSCs retain their capacity to differentiate *in vitro* and *in vivo* into neuronal progenitor cells (NPCs) and that the absence of SMARCB1 affects subsequent neuronal differentiation. Our findings are further supported by a recently published study by Terada et al. (2019), who targeted SMARCB1 in hPSCs to generate a model for AT/RT and found that AT/RT cells of origin are undifferentiated cells at a very early developmental stage, before their differentiation into NPCs. Although this study focused mostly on AT/RT formation upon xenograft transplantation, their results also show that the *SMARCB1*^−/−^ cells readily differentiate into NPCs.

Collectively, these findings suggest a complex regulation of neuronal differentiation by SMARCB1, where any deviation from normal SMARCB1 levels may perturb the neuronal differentiation capacity in a different way. In a more general view, the specific difference between partial and complete SMARCB1 LOF regarding neuronal differentiation may reflect a broader dissimilarity between the effect of these modifications on hPSC fate. For example, the discrepancy between the effect of partial SMARCB1 LOF, which was strongly biased toward upregulation of gene expression (Langer et al., 2019), and the effect we observed upon complete LOF, which was biased toward downregulation of gene expression (∼240 upregulated genes and ∼440 downregulated genes).

Finally, we found that SMARCB1 expression is essential for cell-cell and cell-ECM interactions in hPSCs. The effect of SMARCB1 LOF on these interactions may explain, at least in part, the abnormal self-renewal and differentiation capacity of the cells. Since these interactions were not reported to be impaired by partial SMARCB1 LOF (Langer et al., 2019), we assume that even low SMARCB1 levels are sufficient to maintain the normal cell-cell and cell-ECM interactions in hPSCs. It was shown previously that SMARCB1 plays a vital role in the maintenance of normal cell adhesion and morphology in cancer and transformed cells, such as 293, MCF7 (Caramel et al., 2008), and NCCIT (You et al., 2013) cells. In addition, Darr et al. (2014) found that re-expression of SMARCB1 in RT cells leads to upregulation of GO terms related to cell adhesion, extracellular space, and integrin pathway. These observations strongly support our results, which provide the first indication for the role of SMARCB1 in cell interactions in hPSCs. Our results further show that SMARCB1 LOF impairs the unique actin fence organization of actin fibers in hPSC colonies. This finding is also supported by previous studies in RT cell lines, which reveal the interplay between SMARCB1 and actin fibers organization (Caramel et al., 2008; Darr et al., 2015; Medjkane et al., 2004). Yet, by contrast to RT cells (Darr et al., 2015), it appears that in hPSCs SMARCB1 expression is not required for the initial adhesion to the plate. Importantly, the observation that SMARCA4 LOF leads to downregulation of genes involved in cell adhesion (Zhang et al., 2014) shows that other SWI/SNF core subunits also participate in the regulation of cell-cell and cell-ECM interactions in hPSCs. On the other hand, the fact that the morphological changes caused by SMARCA4 LOF were completely different from those seen upon SMARCB1 LOF indicates a unique role for each of these subunits in cell adhesion.

Our results suggest that, in the case of SMARCB1, the regulation of cell interactions is mediated at least in part via the WNT pathway, as SMARCB1 LOF leads to downregulation of several genes which are part of this pathway as well as of activated β-catenin. This conclusion is further supported by the partial rescue of the morphological phenotype by the WNT activator CHIR99021. Interestingly, SMARCB1 LOF during mouse limb morphogenesis, as well as in RT cell lines, results in the opposite effect, i.e., abnormal activation of the WNT pathway (Mora-Blanco et al., 2014). This dissimilar effect of SMARCB1 LOF suggests a complex and cell-dependent regulation of the WNT pathway by SMARCB1. Finally, it has been shown that a catenin-F-actin-cadherin complex is required for normal cell adhesion (Pieters and van Roy, 2014; Yonemura et al., 1995, 2010). Our observations regarding the effect of SMARCB1 LOF on cell adhesion, actin organization, and β-catenin activation suggest a possible regulation of this complex by SMARCB1 in hPSCs.

In summary, the role of the SWI/SNF complex and its SMARCB1 subunit in epigenetic regulation of diverse cell types has been extensively studied during the last years. In the context of hPSCs, it has been shown that the SWI/SNF complex binds to enhancers and super-enhancers of genes, which control the self-renewal and differentiation of the cells (Langer et al., 2019; Zhang et al., 2014). In the current study, we explored the effects of SMARCB1 complete LOF and overexpression on hPSCs. Overall, the results of our study, along with Langer et al.'s and Terada et al.'s results, show that SMARCB1 levels have to be accurately regulated in order to maintain the self-renewal capacity and pluripotency of hPSCs and that any deviation from normal SMARCB1 expression levels affects hPSC fate in a different manner. We further define, for the first time, SMARCB1 as a critical regulator of normal cell-cell and cell-ECM interactions of hPSCs.

## Experimental Procedures

### Establishment of SMARCB1 Conditional Expression System

Establishment of KI cells: SMARCB1 cDNA was cloned into AAVS1-TRE3G-EGFP donor plasmid. A gRNA targeting AAVS1 locus was designed and cloned into pSpCas9(BB)-2A-GFP plasmid. The donor and the pSpCas9 plasmids were cotransfected into hPSCs. Puromycin-resistant clones were selected and subjected to PCR validation of the appropriate integration.

Establishment of KIKO cells: gRNAs targeting sequences upstream and downstream SMARCB1 exon 2 (see Figure S1) were designed and cloned into pSpCas9(BB)-2A-GFP plasmid. KI cells were cotransfected with both plasmids. Single-cell-derived clones were obtained after GFP FACS sorting and subjected to PCR analysis to detect exon 2 excision. For a detailed description, see Supplemental Experimental Procedures.

To maintain normal SMARCB1 levels during the derivation and expansion of KIKO cells, the cells were grown in the presence of low Dox concentration (12 ng/mL Dox, Sigma-Aldrich). For complete LOF experiments, the Dox was withdrawn from the KIKO cells (see Figure 1A). For gain-of-function experiments, KI cells were grown in the presence of high Dox concentration (50 ng/mL).

### Statistics

All data were generated from at least three biological replicates performed independently. p values were calculated by either Student's t tests or one-way ANOVA with adjusted p value for multiple testings using GraphPad Prism software. For additional information regarding the statistical analyses, see figure legends.

For additional procedures, see Supplemental Experimental Procedures.

### Data and Code Availability

The RNA-seq data have been deposited in NCBI's GEO repository. GEO: GSE158842.

## Author Contributions

I.C-G. designed the study, performed the experiments, and analyzed the data. E.L. performed the experiments. L.A. designed the study, analyzed the data, and wrote the manuscript. O.Y performed the RNA-seq. H.W.B-A. performed the bioinformatics analysis. A.U. conceived and designed the study, analyzed the data, and wrote the manuscript.

## Supplemental Information

Document S1. Supplemental Experimental Procedures, Figures S1 and S2, and Table S1

Document S2. Article plus Supplemental Information
