# Supplementary material for: Human Pluripotent Stem Cell Fate Regulation by SMARCB1
Source: Stem Cell Reports. 2020 Oct 29;15(5):1037–46. doi: 10.1016/j.stemcr.2020.10.002 (PMC7664050; doi:10.1016/j.stemcr.2020.10.002)
Supplement: Document S1. Supplemental Experimental Procedures, Figures S1 and S2, and Table S1 [file mmc1.pdf]

Stem Cell Reports, Volume *15*

Supplemental Information

Human Pluripotent Stem Cell Fate Regulation by SMARCB1

Ilana Carmel-Gross, Etgar Levy, Leah Armon, Orly Yaron, Hiba Waldman Ben-Asher, and Achia Urbach

Supplemental Information

Supplemental Figures

Figure S1

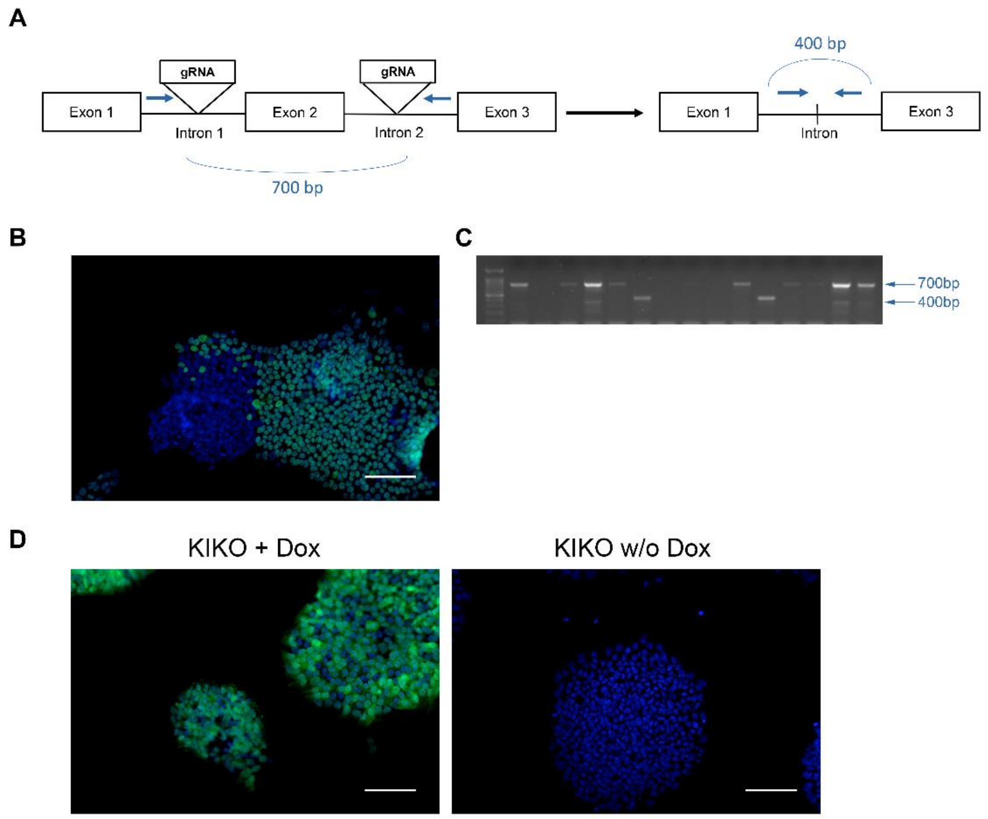

Figure S1 – Targeting *SMARCB1* by the CRISPR/Cas9 system. A. The combination of two gRNAs directed upstream and downstream to exon 2 leads to the excision of the entire exon. The blue arrows represent PCR primers flanking the sequences targeted by the gRNAs. The removal of the exon, results in a decrease in the PCR product size from 700 bp to 400 bp. **B.** SMARCB1 immunostaining of hPSC population following introduction of the CRISPR system. Note the absence of SMARCB1 from some of the cells. Green – SMARCB1. Blue – DAPI. **C.** Representative PCR results (using the abovementioned primers) showing WT, heterozygous and homozygous hPSC clones. **D.** Immunostaining of single cell derived KIKO clones from hiPSCs in the presence of Dox or 96h following Dox withdrawal. Green – SMARCB1. Blue – DAPI. Scale bar: 100μm.

Figure S2

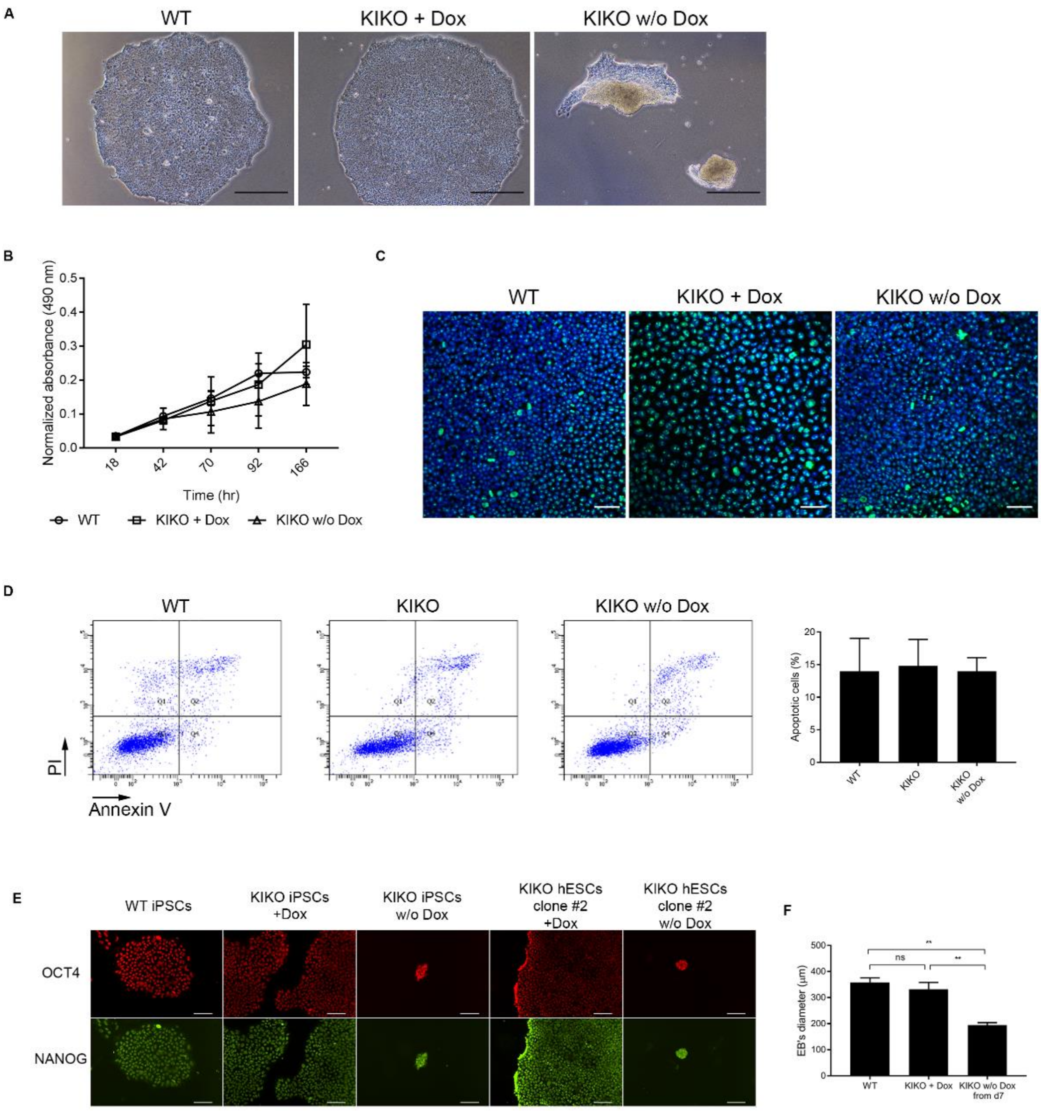

Figure S2 – SMARCB1 complete LOF effect on hPSC fate. A. Phase-contrast images of representative SMARCB1 LOF and control hiPSC colonies. **B.** MTS assay for cell viability and proliferation for SMARCB1 LOF and control hESCs. No statistical significant differences were found at any of the time points. Statistical analysis was done by two-way ANOVA, n=3. **C.** Ki67 immunostaining of SMARCB1 LOF and control hESCs. Green - Ki67, Blue – DAPI. Scale bar: 100 µm **D.** Apoptosis assay based on AnnexinV. Representative FACS results for each sample and quantification of three biological repeats are presented. No statistical significant difference was found according to one-way ANOVA. **E.** Representative Oct4 and Nanog immunostaining results for KIKO iPSCs and an additional clone of KIKO hESCs upon single cell passaging. Scale bar: **F.** Size measurement of EBs derived from control or SMARCB1 LOF cells. Dox withdrawal from d0 resulted in very few EBs, therefore size measurement was not applicable for these cells. One-way ANOVA with Tukey post-test. P<0.001.

Supplemental Tables

Table S1 – primers used for qRT-PCR

Primer name	Forward	Reverse
MIXL1	GGCGTCAGAGTGGGAAATCC	GCAGTTCACATCTACCTCAAGAG
PAX6	TCTTTGCTTGGGAAATCC	CTGCCCGTTCAACATCCTTAG
CDX2	GGCAGCCAAGTGAAAACCAG	GGTGATGTAGCGACTGTAGTGAA
EOMES	CCCACTACAATGTGTTCGTAGAG	CATTTTGTTGCCCTGCATGTT
FOXA2	GGTGTCTGAGGAGTCGGAGA	CCTCGGGCTCTGCATAGTAG
SOX17	AAGGGCGAGTCCCGTATC	TTGTAGTTGGGGTGGTCCTG
NEUROG2	CGTCCTCCTCCGTGTCCTCCAATTCCACCT	AAGAGAAAGGGGAGGAGCGTCAGTCCGCTC
NR4A2	AGTCTGATCAGTGCCCTCGT	TATGCTGGGTGTCATCTCCA
T	TCAGCAAAGTCAAGCTCACCA	CCCCAACTCTCACTATGTGGATT
GATA6	AGAAACGCCGAGGGTGAAC	AGTTGGAGTCATGGGAATGGAA
GDNF	CTGCCTGGTGCTGCTCCACA	AGCTGCAGCCTGCCGATTCC
NCAM1	GATGCGACCATCCACCTCAA	TCTCCGGAGGCTTCACAGGTA
βACTIN	CACCTTCTACAATGAGCTGCGTGTG	ATAGCACAGCCTGGATAGCAACGTAC

Supplemental Experimental Procedures

Establishment of SMARCB1 conditional expression system

Establishment of KI cells: *SMARCB1* cDNA was cloned into AAVS1-TRE3G-EGFP donor plasmid. kindly donated by Dr. Su-Chun Zhang (Qian et al., 2014) using MluI and SalI restriction enzymes to replace the EGFP sequence. gRNA (GTCCCCTCCACCCCACAGTG) targeting AAVS1 locus was designed and cloned into pSpCas9(BB)-2A-GFP plasmid (Addgene #48138) as described in (Ran et al., 2013). The donor and the pSpCas9 plasmids were cotransfected into hPSCs using LipofectaminStem (Thermo). Puromycin resistant clones (indicating donor sequence integration into an active gene, most probably *AAVS1* locus upon homologous recombination) were selected and subjected to PCR validation of the appropriate integration, using the following primers: set 1: forward: CAAACAGCATAAGCTGGTCAC reverse: CTGACGCTCTTGACGATTTTGAC set 2: forward: GAAGAGTTCTTGCAGCTCG reverse: CTTTGAGCTCTACTGGCTTC.

Establishment of KIKO cells: gRNAs targeting sequences upstream (CTGCCGAAAGCGTGGCGCCT) and downstream (AGCCTTGGCCTTAGTCGGGC) SMARCB1 exon 2 (see **Figure S1**) were designed and cloned into pSpCas9(BB)-2A-GFP plasmid (addgene #48138) as described in (Ran et al., 2013). KI cells were cotransfected with both plasmids. Single cell derived-clones were obtained following GFP FACS sorting and subjected to PCR analysis to detect exon 2 excision using the following primers: forward primer: CCTTTCGGAACTTGGTTCTGTTG. reverse primer: GATGTGCTCCAGGAAGCAAG as described in Figure S1.

Cell culture

hESCs (HUES13) and hiPSCs were grown feeder-free on vitronectin (PeproTech) coated plates in mTeSR1 medium (STEMCELL Technologies). Cells were passaged either with ReLeSR (STEMCELL Technologies) or accutase (Sigma) for single cell passaging. The cells were treated with 10uM ROCK inhibitor (Y27632, PeproTech) overnight upon single cell passaging.

Single cell passaging assay

Dox was withdrawal for 72h prior to the assay. Cells were harvested as single cells and plated at 100,000cells/well of 12 well plate. The morphology and cell number were evaluated 96h post the single cell seeding. For WNT pathway experiment CHIR99021 (2μM, Tocris) was added starting from the day of Dox withdrawal.

Reprogramming of somatic cells into hiPSCs.

Fibroblasts obtained from hPSC (CSES7 line) derived teratoma, were reprogrammed into hiPSCs using the CoMIP plasmid (Addgene #63727) according to the protocol published by (Diecke et al., 2015). CSES7 hPSCs were kindly provided by Prof. Benvenisty, The Hebrew University, Israel.

Western Blot assay

Western blot was performed according to the usual procedures. The following antibodies were used: SMARCB1 (Abcam, ab58209, 1:500), OCT4 (Santa Cruz Biotechnologies, sc-5279, 1:500), Nanog (Abcam, ab80892, 1:1000 or R&D Systems, AF1997, 1:200), activated β-catenin (Millipore, 05-665, 1:1000), α-Tubulin (Cell Signaling, 2144, 1:1000) and GAPDH (Millipore, ABS16, 1:1000).

Immunoflurescence

Cells were fixed with 4% paraformaldehyde and permeabilized for 5 min with 0.1% Triton in PBS. Blocking was performed with 5% goat serum, 0.05% Triton X100 in PBS. Primary antibodies were applied for two hours in RT, washed and incubated with fluorophore conjugated secondary antibodies for one hour at RT. Nuclei were stained with DAPI. The following primary antibodies were SMARCB1 (Abcam, ab192864, 1:500), NF-H (Sigma, N4142, 1:250), Oct4 (Santa Cruz Biotechnologies, sc-5279, 1:500), NANOG (R&D systems, AF1997, 1:100), and Ki67 (Invitrogen, 14-5698-82, 1:500). Actin fibers were stained with Phalloidin-iFluor488 (Abcam, 1:1000).

qRT-PCR

Total RNA was extracted using a RiboEx Total RNA purification solution (GeneAll), followed by reverse transcription using iScript cDNA Synthesis Kit (BioRad). qRT-PCR was performed using FastStart Universal SYBR Green Master Mix (Roche). β-actin was used for normalization. For primers list see **Table S1.**

Aggregation (EBs formation) assay

To generate uniform size cell aggregates the cells were seeded in agarose microwells as follows: Agarose and NaCl solution was poured into 256 MicroTissues 3D Petri Dish micro-mold (Sigma-Aldrich, Z764000) to prepare microwells as described in (Birenboim et al. 2013). Cells were harvested with Accutase (Sigma-Aldrich) and plated into the 256 microwells (750,000 cells per microwell plate, ~3000 cells per each well). The cells were grown in EBs medium - DMEM/F12 (Biological Industries), 10% KOSR (Thermo Fisher Scientific), 1% MEM non-essential amino acids (Biological Industries), 0.1 mM β-mercaptoethanol, 1 mM glutamine (Biological Industries) and 1% Pen/Strep (Biological Industries). EBs grown in molds for two weeks and then transferred to a non-adherent plate and analyzed EB formation at day 30.

Neuronal differentiation

Neuronal differentiation was performed according to (Birenboim et al. 2013) with minor modifications. Cells ($7.5X10^5$) were resuspended in 100μl EBs medium supplemented with 10μM SB431542 (Tocris), 2μM dorsomorphin dihydrochloride (Tocris) and Y27632 (PeproTech). Next, the cells were transferred to microwells (see above). Four days after the initial seeding the medium was changed to EBs medium. At day 14 cells aggregates were transferred to פoly-L-Lysine (Sigma-Aldrich) and laminin (Sigma-Aldrich) coated plate in neural induction medium (DMEM/F12, 1mM L-Glutamin, 1% Pen/Strep, 2% B27 (Thermo Fisher Scientific), 10ng/ml NGF (Alomone Labs), 10ng/ml NT-3 (Alomone Labs), 10ng/ml BDNF (Alomone Labs) and 10ng/ml GDNF (PeproTech).

Endodermal and ectodermal differentiation

STEMDiff Trilineage Differentiation Kit (Stemcell Technologies) was used for endodermal and ectodermal differentiation according to manufacturer's instructions. For SMARCB1 LOF, Dox was removed from KIKO cells one day before seeding for endoderm differentiation and one day after seeding for ectodermal differentiation. Overall the KIKO cells were without Dox for seven days at the end of each protocol or with Dox for the entire period as control.

Mesodermal differentiation

Cells were seeded at low density ($9X10^4$ cells/12well) two days prior to mesodermal differentiation induction. Then, the cells were cultured for 2 days in RPMI1640 medium (Biological Industries) supplemented with B27 without insulin (Thermo Fisher Scientific) and 8 μM CHIR99021 (Tocris). Next, the medium was changed to RPMI1640 supplemented with B27 without insulin alone, for two additional days. For SMARCB1 LOF experiment Dox was removed from KIKO cells one day before cell seeding.

Teratoma formation

Cells (2X 10cm plates 90% confluent) were resuspended in 100μl mTeSR and mixed with 100μl Matrigel (Corning). Then the cells were subcutaneously injected into SCID mice (NOD.CB17-Prkdc-scid, Envigo). Teratomas were harvested 8 weeks following the transplantation, fixed with 4% formaldehyde and embedded in paraffin. The teratomas were stained by standard H&E staining protocols. All animal procedures were conducted according to animal care guidelines approved by the Institutional Animal Care and Use Committee at Bar Ilan University (approval #11-02-2018).

Apoptosis assay

Apoptosis analysis was performed using MEBCYTO-Apoptosis Kit (MBL) according to manufacturer's instructions for FACS analysis. For SMARCB1 LOF Dox was removed seven days prior to the analysis.

Proliferation assay

Proliferation assay was using the CellTiter 96® AQueous One Solution Cell Proliferation Assay (MTS) (Promega). The starting seeding density was 1000 cells/well (96 well plate). For SMARCB1 LOF experiment Dox removed from KIKO cells at seeding day.

ECM adhesion assay

For cellular adhesion assay we used the CytoSelect™ 48-Wll Cell Adhesion Assay (ECM Array, Colorimetric, Cell Biolabs), According to the manufacturer's instructions. Dox was withdrawn 96h prior to the assay. Cells were harvested as single cells. 80,000 cells/well were plated. The adhesion was evaluated 90min after cell seeding. The vitronectin experiment was done separately by coating regular 48 well plate with vitronectin following the same analysis as for the other ECM proteins.

RNAseq

RNA quality control - For QC of purified RNA, absorbance ratios A260:A280 and A260:A230 were assessed with NanoDrop 2000. The integrity of RNA was evaluated based on RIN acquired via capillary gel electrophoresis performed using Agilent 4200 TapeStation in combination with Agilent RNA ScreenTape System (Agilent Technologies). All RNA samples went through DNase Treatment Kit (Qiagen) before proceeding to the next step.

PolyA selection and library preparation - For library preparation NEBNext RNA ultra II RNA library preparation kit (NEB) was used. All RNA samples underwent PolyA selection following the manufacturers' protocols. Samples were multiplexed using suitable molecular barcodes and resulting cDNA pools were processed according to the NextSeq System Denature and Dilute Libraries guide. (Illumina). Quantification and quality control of the libraries were done using Qubit fluorimeter and Agilent 4200 TapeStation.

Next-generation sequencing - Single-read sequencing of the libraries with a read length of 75 was performed with NextSeq 500 Sequencing System using NextSeq 500/550 High Output v2 kit (75 cycles) (20024906 Illumina). PhiX Control v3 (Illumina) was added at 1% to all pools as an internal control before the sequencing.

Bioinformatics analysis – Sequenced reads were mapped to the human reference genome sequences (hg19) using STAR. The aligned reads were quantitated by Htseq. The normalization and differentially expressed genes test were implemented by DESeq2. An arbitrary cutoff of at least 2-fold and p-value adjusted for multiple testing < 0.05 were chosen to define a differentially expressed gene. Geneanalytics tool was used for enrichment analysis on the differentially expressed genes between each group.

The raw data have been deposited in the NCBI Sequence Read Archive (SRA) with SRA accession number: PRJNA574055.

References

Diecke, S., Lu, J., Lee, J., Termglinchan, V., Kooreman, N.G., Burridge, P.W., Ebert, A.D., Churko, J.M., Sharma, A., Kay, M.A., et al. (2015). Novel codon-optimized mini-intronic plasmid for efficient, inexpensive and xeno-free induction of pluripotency. Sci. Rep. *5*, 8081.

Qian, K., Huang, C.-L., Chen, H., Blackbourn, L.W., Chen, Y., Cao, J., Yao, L., Sauvey, C., Du, Z., Zhang, S.-C., et al. (2014). A Simple and Efficient System for Regulating Gene Expression in Human Pluripotent Stem Cells and Derivatives. Stem Cells *32*, 1230–1238.

Ran, F.A., Hsu, P.D., Wright, J., Agarwala, V., Scott, D.A., and Zhang, F. (2013). Genome engineering using the CRISPR-Cas9 system. Nat. Protoc. *8*, 2281–2308.
