# Supplementary material for: Human Pluripotent Stem Cell Fate Regulation by SMARCB1
Source: Stem Cell Reports. 2020 Oct 29;15(5):1037–46. doi: 10.1016/j.stemcr.2020.10.002 (PMC7664050; doi:10.1016/j.stemcr.2020.10.002)
Supplement: Document S2. Article plus Supplemental Information [file mmc2.pdf]

Stem Cell Reports
Report

OPEN ACCESS

Human Pluripotent Stem Cell Fate Regulation by SMARCB1

Ilana Carmel-Gross,[1] Etgar Levy,[1] Leah Armon,[1] Orly Yaron,[1] Hiba Waldman Ben-Asher,[1] and Achia Urbach[1,*]
[1]The Mina and Everard Goodman Faculty of Life Sciences, Bar-Ilan University, Ramat Gan 5290002, Israel
*Correspondence: achia.urbach@biu.ac.il
https://doi.org/10.1016/j.stemcr.2020.10.002

SUMMARY

Epigenetic regulation by the SWI/SNF complex is essential for normal self-renewal capacity and pluripotency of human pluripotent stem cells (hPSCs). It has been shown that different subunits of the complex have a distinct role in this regulation. Specifically, the SMARCB1 subunit has been shown to regulate the activity of enhancers in diverse types of cells, including hPSCs. Here, we report the establishment of conditional hPSC lines, enabling control of SMARCB1 expression from complete loss of function to significant overexpression. Using this system, we show that any deviation from normal SMARCB1 expression leads to cell differentiation. We further found that SMARCB1 expression is not required for differentiation of hPSCs into progenitor cells, but rather for later stages of differentiation. Finally, we identify SMARCB1 as a critical player in regulation of cell-cell and cell-ECM interactions in hPSCs and show that this regulation is mediated at least in part by the WNT pathway.

INTRODUCTION

The SWI/SNF chromatin remodeling complex controls the chromatin structure by nucleosome mobilization. The human SWI/SNF complex contains a single ATPase (SMARCA2 also known as BRM or SMARCA4 also known as BRG1), 3 core subunits, and 7 to 15 additional accessory subunits. The specific composition of the subunits varies between different tissues (for review, see Masliah-Planchon et al., 2014). The chromatin remodeling capacity of the complex enables tight regulation of gene expression. Therefore, it plays an important role in regulation of many cellular processes (Masliah-Planchon et al., 2014), including during preimplantation embryonic development (Kim et al., 2001; Klochendler-Yeivin et al., 2000). In agreement with its role during early embryonic development, it has been shown that SMARCA4 (Kidder et al., 2009), the core subunits ARID1A/B (BAF250a/b) (Gao et al., 2008; Yan et al., 2008), SMARCB1 (Schaniel et al., 2009), and the specific composition of the accessory subunits (Ho et al., 2009) are essential to maintain the self-renewal capacity and pluripotency of mouse embryonic stem cells (mESCs). These observations raise the question of whether SMARCB1 plays a similar role also in human pluripotent stem cells (hPSCs), which are fundamentally different from naive mESCs (Nichols and Smith, 2009; Yilmaz and Benvenisty, 2019). To address this question, Zhang et al. (2014) compared the role of several SWI/SNF subunits between mESCs and hPSCs. This analysis revealed several significant differences between the composition and function of the various SWI/SNF subunits in mouse versus human hPSCs.

The SWI/SNF core subunit SMARCB1 (also known as INI1, SNF5, and BAF47) has been shown to control recruitment of the SWI/SNF complex to enhancers and bivalent promoters, thus regulating their activation (Alver et al., 2017; Nakayama et al., 2017; Wang et al., 2017). The fact that SMARCB1 loss of function (LOF) is the sole mutation found in the vast majority of rhabdoid tumors (highly aggressive pediatric tumors arising mainly in the brain (AT/RT) and kidneys (for review see [Lee et al., 2012; Masliah-Planchon et al., 2014]) emphasizes its critical role in cell fate epigenetic regulation.

Recently, Langer et al. (2019) have found that SMARCB1 suppresses the activity of hPSC super-enhancers during neuronal differentiation and thus enables the pluripotent cells to differentiate toward this lineage. This study provides a novel and important insight into the role of SMARCB1 in hPSCs. Yet, it is based on partial downregulation of the gene (~80% reduction compared with normal hPSCs). Therefore, a complementary system of SMARCB1 complete LOF is required to fully understand the involvement of SMARCB1 in hPSC fate regulation.

Here, we report the establishment of human embryonic stem cell (hESC) and human induced pluripotent stem cell (hiPSC) lines enabling the control of SMARCB1 expression levels ranging from null expression to a significant overexpression. We found that both SMARCB1 LOF and SMARCB1 overexpression impair the self-renewal capacity of the cells. We further show that SMARCB1 complete LOF affects the differentiation capacity of hPSCs; however, in a different manner than partial SMARCB1 LOF. Finally, our data provide the first indication for the role of SMARCB1 in the maintenance of normal cell-cell and cell-extracellular matrix (ECM) interactions in hPSCs and reveal the involvement of the WNT pathway in these processes.

RESULTS

Establishment of Conditional SMARCB1 LOF hPSC Lines

To study the role of SMARCB1 in hPSCs, we first aimed to target *SMARCB1* using the CRISPR-Cas9 system with two

This is an open access article under the CC BY license (http://creativecommons.org/licenses/by/4.0/).

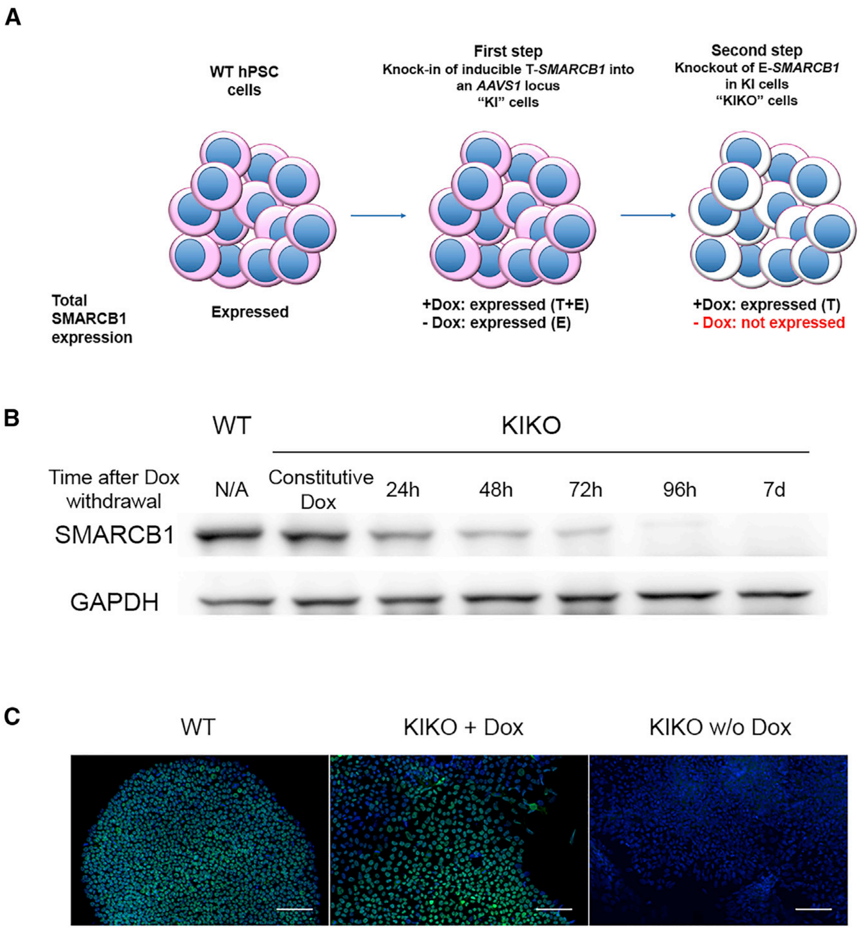

Figure 1. Conditional SMARCB1 LOF in hPSCs

(A) Scheme describing the two-step approach for SMARCB1 conditional knockout in hPSCs. In the presence of Dox, the KI cells express both the endogenous (E) and the transgenic (T) SMARCB1, while the KIKO cells express only the transgenic SMARCB1. In the absence of Dox, there is no expression of SMARCB1 in the KIKO cells.

(B) Western blot analysis of hESC wild-type (WT) and KIKO cells in the presence of Dox and at different time points after Dox withdrawal.

(C) Immunostaining of single-cell-derived KIKO clones from hESCs in the presence of Dox or 96 h after Dox withdrawal. Green, SMARCB1; blue, DAPI. Scale bar, 100 μm.

gRNAs directed upstream and downstream of *SMARCB1* exon 2 (Figure S1A). We first verified this system at the population level and found, as expected, that the transfected cells show patches of SMARCB1-negative cells (Figure S1B). Next, we screened by PCR single-cell-derived clones and found that 23% (15/64) of the clones were heterozygous for exon 2 deletion (*SMARCB1*$^{+/-}$). Notably, however, we did not find even a single *SMARCB1*$^{-/-}$ clone. These results suggest that SMARCB1 LOF might have a negative effect on the growth/morphology of hPSCs. Therefore, we established a conditional SMARCB1 LOF system based on a two-step approach. First, we knocked in a *SMARCB1* conditional (Tet-On) overexpression cassette into the AAVS1 locus of hPSCs (herein KI cells). Next, we targeted the endogenous *SMARCB1* in the KI cells in the presence of low doxycycline (Dox) concentration (12 ng/mL) to maintain normal SMARCB1 levels. This strategy enabled us to isolate clones of hESCs and of hiPSCs with a homozygous deletion of exon 2 (Figure S1C) in reasonable efficiency (3/58 and 4/17 homozygous clones in hESC and hiPSC lines, respectively). These clones (herein KIKO cells, knockout on the background of knockin) retain normal levels of SMARCB1 expression in the presence of Dox (herein KIKO + Dox) but completely fail to express the gene within 4 days upon Dox withdrawal (herein KIKO w/o Dox) (Figures 1A–1C and S1D).

Deviations from Normal SMARCB1 Levels Affect the Self-Renewal Capacity of hPSCs

Previous studies revealed that downregulation of SMARCB1 (Langer et al., 2019) or other SWI/SNF core subunits (Zhang et al., 2014) leads to rapid upregulation of differentiation markers, while pluripotent genes, such as *OCT4* and *NANOG* are still normally expressed. To examine if SMARCB1 complete LOF has the same effect on hPSCs, we performed RNA sequencing (RNA-seq) of KIKO and control cells 7 days after Dox withdrawal (3 days after complete SMARCB1 LOF). Our RNA-seq analysis revealed ~240 upregulated and ~440 downregulated genes upon SMARCB1 LOF. Gene ontology (GO) annotation of the RNA-seq data indicates a rapid upregulation of biological processes related to multicellular organism development, and

specifically neuronal development (Figure 2A). As in the abovementioned studies, these changes in gene expression were not accompanied by downregulation of pluripotent markers, such as OCT4 and NANOG (Figures 2B and 2C).

Next, we studied whether SMARCB1 overexpression also affects the self-renewal capacity of the cells. For this purpose, we cultured the KI cells for 14 days in the presence of high Dox concentration (50 ng/μL), which led to a significant increase in SMARCB1 levels (Figures 2D and 2E). This upregulation led to cell differentiation, as demonstrated by the significant downregulation of OCT4 and NANOG (Figures 2D and 2E), morphological changes (Figure 2F), and GO annotation of the upregulated genes found by RNA-seq (Figure 2G). Notably, the downregulation of the pluripotency markers appeared already after a short time (7 days) of Dox treatment (data not shown). Overall, these results, together with the previous observations (Langer et al., 2019) regarding the effect of partial SMARCB1 downregulation, indicate that SMARCB1 has to be precisely regulated in hPSCs in order to maintain their self-renewal capacity.

SMARCB1 Complete LOF Affects the Differentiation Capacity of hPSCs

To study the effect of SMARCB1 complete LOF on the pluripotency of the cells, we first evaluated the capacity of the cells to differentiate *in vitro* into the three germ layers using a direct differentiation method. We found that the SMARCB1 LOF cells retained their capacity to differentiate into the mesoderm, ectoderm, and endoderm lineages similar to the control cells (Figure 3A). These results indicate that SMARCB1 expression is not required for differentiation of the hPSCs into cells of the three germ layers. Notably, the expression of *PAX6* and *NEUROG2* upon ectodermal differentiation (Figure 3A) suggests that, by contrast to partial SMARCB1 LOF (Langer et al., 2019), a complete silencing of the gene does not abrogate hPSC neuronal differentiation capacity. To further confirm this observation, we used a direct neuronal differentiation protocol (Birenboim et al., 2013). Indeed, a gene expression analysis revealed that SMARCB1 LOF cells successfully differentiated into the neuronal lineage (Figure 3B). Interestingly, however, the neuronal differentiation seems to be impaired in these cells (Figure 3C). Overall, these results suggest that, while SMARCB1 is not essential for the differentiation of the cells into progenitor cells of the three germ layers, its expression is required for the subsequent differentiation at least into the neuronal lineage. To validate this assumption, we performed a teratoma formation assay. The teratomas derived from the SMARCB1 LOF cells contained mesodermal derivatives, such as bone and cartilage (Figure 3D), but mature ectodermal and endodermal cells were scarcely detected in these teratomas except for immature neural tube structures (Figure 3D, bottom middle). Moreover, patches of undifferentiated cells were found across these teratomas (Figure 3D, bottom middle and right). The fact that these patches were OCT4 negative (data not shown) indicates that they comprise cells that started to differentiate, but failed to complete their differentiation. These *in vivo* results strongly support the assumption that SMARCB1 is required for maturation of the ectodermal lineage (and probably also for endodermal derivatives).

SMARCB1 Is Required for Cell-ECM and Cell-Cell Interactions of hPSCs

In addition to the abovementioned phenotypes, the most prominent effect of SMARCB1 LOF was significant morphological changes of the hPSC colonies. Three days after complete SMARCB1 silencing, the cells started to grow as multilayered 3D colonies instead of spreading horizontally as monolayer colonies (Figures 4A and S2A). These morphological alterations were not accompanied by significant changes in the proliferation (Figures S2B and S2C) and apoptosis of the cells (Figure S2D). Moreover, a single-cell passaging of the SMARCB1 LOF cells significantly abrogated their capacity to generate typical hPSC colonies. Specifically, the single-cell-derived colonies were small and compacted, and many of them failed to stay attached to the plate (Figure 4B), but retained the expression of pluripotency markers (Figures 4C and S2E). Altogether, these observations suggest that SMARCB1 LOF affects the capacity of hPSCs to establish normal interactions with the ECM. Indeed, RNA-seq analysis revealed that pathways and biological processes related to cell adhesion and ECM organization are significantly downregulated in the absence of SMARCB1 expression (Figure 4D). This effect of SMARCB1 LOF is not due to inability of the mutated cells to adhere to the ECM as evident by cell adhesion assay (Figure 4E). Rather, it is probably the result of a failure to maintain the interactions between the cells and the ECM. A unique organization of actin fibers into ventral stress fibers called actin fence is known to regulate hPSC-ECM interactions (Närvä et al., 2017; Stubb et al., 2019). To explore whether this organization is affected by SMARCB1 LOF, we stained the cells with phalloidin. Remarkably, the SMARCB1 LOF cells failed to establish this unique actin fence organization (Figure 4F). Overall, these observations demonstrate an important role of SMARCB1 in the regulation of the interaction between hPSCs and the ECM.

To explore if SMARCB1 is required also for cell-cell interaction in hPSCs, we studied their aggregation capacity by embryonic bodies (EBs) formation assay. By contrast to the control cells, the SMARCB1 LOF cells (Dox withdrawal at day 0 of the assay) almost completely failed to generate

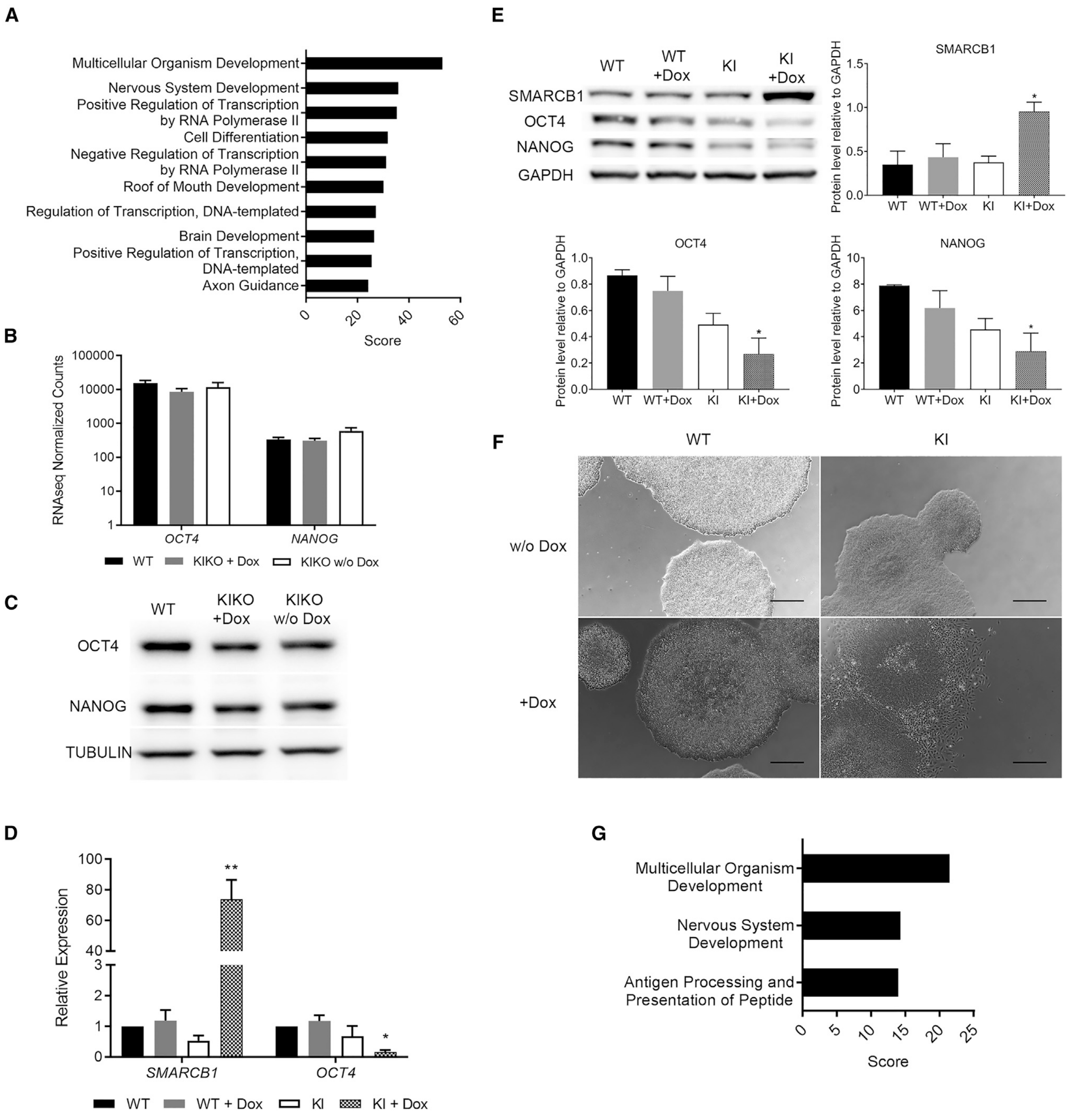

Figure 2. The Effect of SMARCB1 Misregulation on Self-Renewal Capacity of hPSCs
(A) Top high scored gene ontology (GO) terms of genes upregulated upon SMARCB1 complete LOF.
(B and C) OCT4 and NANOG levels in controls and SMARCB1 LOF cells as determined by RNA-seq (B) and western blot analysis (C).
(D) qRT-PCR analysis for *SMARCB1* and *OCT4* in controls and SMARCB1 overexpressing cells.
(E) Representative image and quantification of western blot analysis for SMARCB1, OCT4, and NANOG levels upon SMARCB1 overexpression.
(F) Phase-contrast images of representative hESC colonies. Scale bar, 500 μm.
(G) High scored GO terms of genes upregulated upon SMARCB1 overexpression.
All quantitative data are represented as means of three biological replicates. qRT-PCR data are normalized to WT. $^{*}p < 0.05$, $^{**}p < 0.001$. Statistical analysis was performed using multiple t tests with false discovery rate (FDR) adjustment (D) and one-way ANOVA with Dunnett's post hoc test (E).

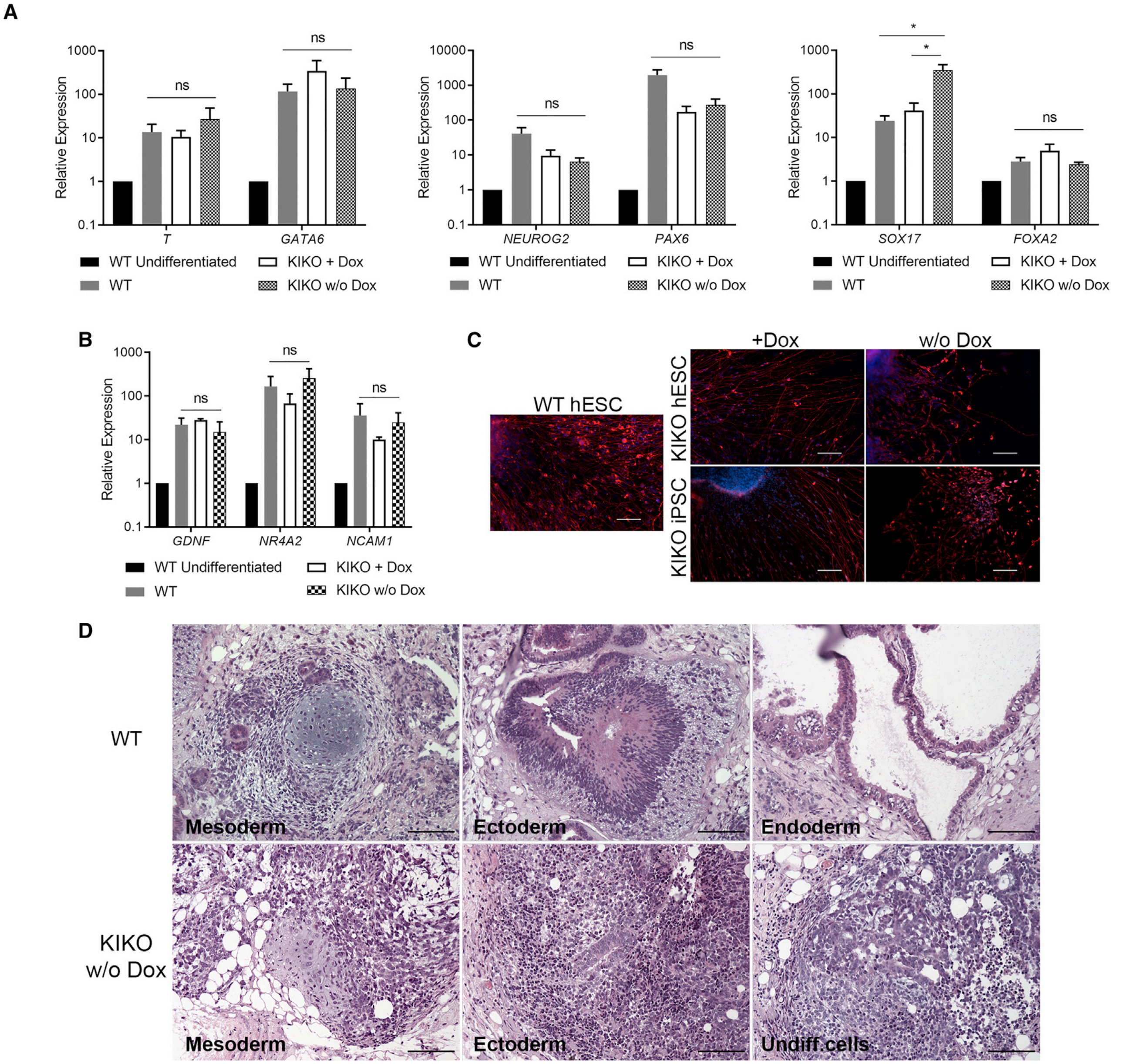

Figure 3. The Effect of SMARCB1 LOF on hPSC Differentiation

(A) qRT-PCR analysis of mesodermal (left), ectodermal (middle), and endodermal (right) genes upon direct differentiation.

(B) qRT-PCR analysis of neuronal markers upon direct neuronal differentiation. qRT-PCR data in (A and B) are represented as mean of three biological replicates ± SEM. Data are normalized to WT undifferentiated cells. Statistical analysis using one-way ANOVA with FDR correction. ns, not significant. *$p < 0.05$.

(C) Immunofluorescent staining of neurons derived from control and SMARCB1 LOF cells. Red, NF-H; blue, DAPI. Scale bar, 100 μm.

(D) H&E staining of teratomas derived from WT and SMARCB1 LOF hPSCs. Scale bar, 50 μm.

EBs. Moreover, SMARCB1 LOF affected the cell ability to generate EBs even when it was induced at later stage of the assay (Dox withdrawal at day 7 of the assay) (Figures 4G and S2F). These results indicate that SMARCB1 expression is also required for cell-cell interactions in hPSCs.

Finally, our RNA-seq analysis revealed downregulation of several genes related to the WNT signaling pathway (GO:0016055, *Z* score 13.28). In accordance, we found that the SMARCB1 LOF leads to a significant reduction in activated β-catenin levels (Figure 4H). These results suggest

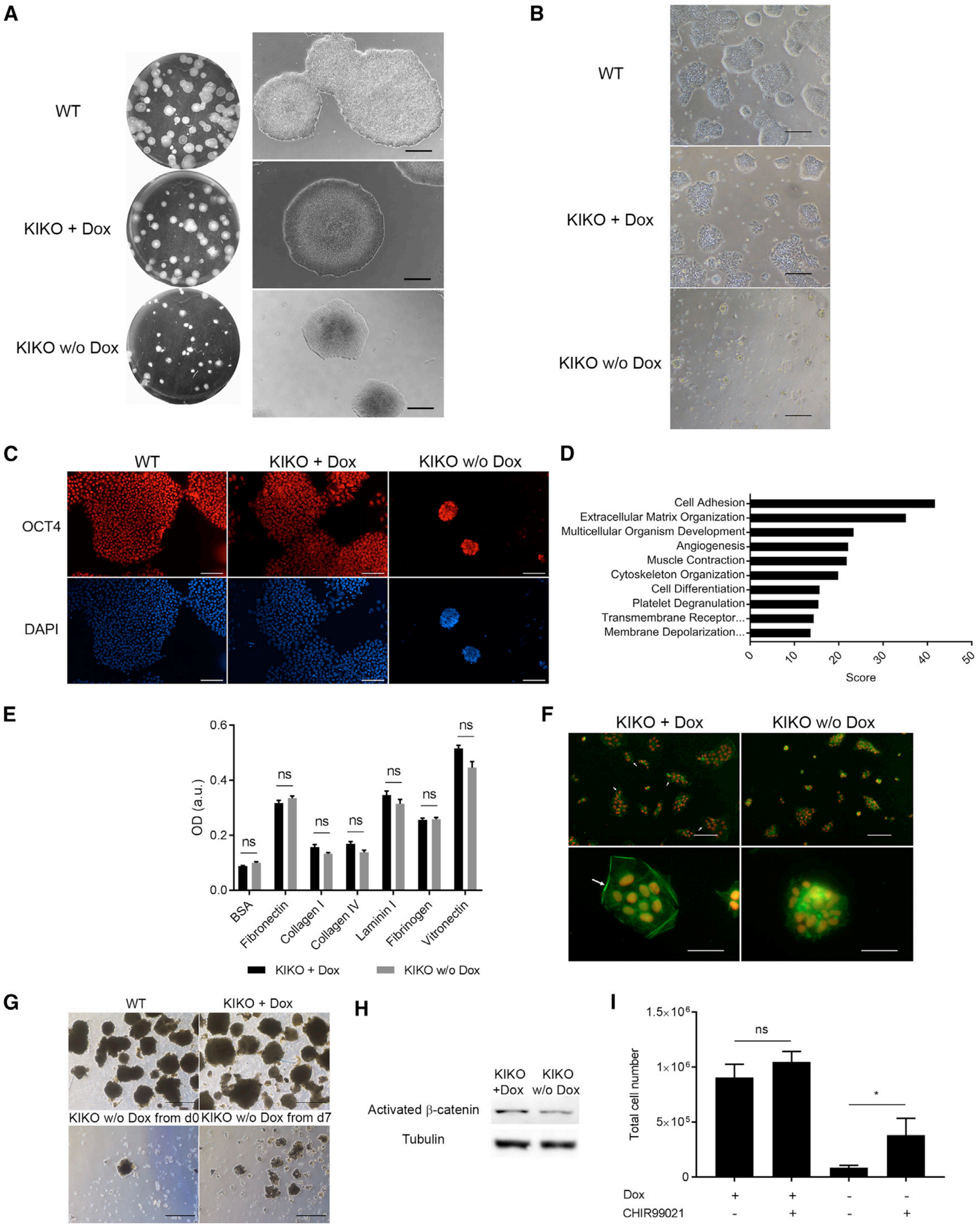

Figure 4. Abnormal Cell-Cell and Cell-ECM Interactions in SMARCB1 Complete LOF hPSCs

(A) Controls and SMARCB1 LOF hPSC colonies. Left: low-magnification, whole-plate imaging. Right: higher magnification of representative colonies. Scale bar, 500 μm.

(B) Controls and complete SMARCB1 LOF colonies 72 h after single-cell plating. Scale bar, 500 μm.

(C) Immunostaining for OCT4, 72 h after single-cell plating. Scale bar, 100 μm.

(D) Top 10 high scored GO terms of genes downregulated upon SMARCB1 complete LOF.

(E) Results of ECM adhesion assay for SMARCB1 LOF and control cells. The assay was done on different types of ECM proteins, as indicated in the figure. BSA was used as a negative control. n = 4 for each condition.

(legend continued on next page)

that the effect of SMARCB1 LOF on hPSC fate is mediated at least in part via inhibition of the WNT pathway. To validate this hypothesis, we explored the effect of the WNT pathway activation by CHIR99021 treatment (2 μM) on SMARCB1 LOF cells. Indeed, this treatment significantly increased the number of SMARCB1 LOF colonies grown attached to the plate upon single-cell passaging (Figure 4I) and thus confirm the interplay between SMARCB1 and WNT pathways in hPSCs.

DISCUSSION

The self-renewal and the differentiation capacity of pluripotent stem cells are tightly epigenetically regulated (Bibikova et al., 2008; Meshorer and Misteli, 2006; Yilmaz and Benvenisty, 2019). Therefore, it is expected that normal expression and function of the SWI/SNF complex will be essential for the maintenance of hPSCs. Here, we show that complete SMARCB1 LOF, as well as SMARCB1 overexpression, leads to hPSC differentiation. These results are in agreement with those of Langer et al. (2019) showing that partial SMARCB1 LOF in hPSCs also leads to hPSC differentiation. A similar effect on hPSC self-renewal capacity was observed upon downregulation of SMARCA4, the SWI/SNF catalytic subunit (Zhang et al., 2014). Yet, this effect is not common for all SWI/SNF core subunits. For example, SMARCC1 depletion has no overt effect on the cells (Zhang et al., 2014).

While the abovementioned results reveal a similar role for SMARCA4 and SMARCB1 in hPSC self-renewal regulation, it appears that these two subunits play a different role in the regulation of hPSC pluripotency. SMARCA4-deficient hPSCs show a reduced capacity to differentiate into the mesodermal lineage but retain their ectodermal differentiation capacity, including neuronal differentiation (Zhang et al., 2014). On the contrary, our results show that SMARCB1 expression is not required for mesodermal differentiation of hPSCs, but rather for neuronal differentiation. Interestingly and counterintuitively, the effect of partial SMARCB1 LOF on neuronal differentiation appears to be more severe than the effect observed upon complete SMARCB1 LOF. Specifically, Langer et al. showed that SMARCB1 functions by silencing of hPSC-specific super-enhancers during the differentiation into the neuronal lineage, and thus SMARCB1 downregulation impairs neuronal induction from hPSCs. In contrast, we found that complete SMARCB1 LOF hPSCs retain their capacity to differentiate *in vitro* and *in vivo* into neuronal progenitor cells (NPCs) and that the absence of SMARCB1 affects subsequent neuronal differentiation. Our findings are further supported by a recently published study by Terada et al. (2019), who targeted SMARCB1 in hPSCs to generate a model for AT/RT and found that AT/RT cells of origin are undifferentiated cells at a very early developmental stage, before their differentiation into NPCs. Although this study focused mostly on AT/RT formation upon xenograft transplantation, their results also show that the *SMARCB1*$^{-/-}$ cells readily differentiate into NPCs.

Collectively, these findings suggest a complex regulation of neuronal differentiation by SMARCB1, where any deviation from normal SMARCB1 levels may perturb the neuronal differentiation capacity in a different way. In a more general view, the specific difference between partial and complete SMARCB1 LOF regarding neuronal differentiation may reflect a broader dissimilarity between the effect of these modifications on hPSC fate. For example, the discrepancy between the effect of partial SMARCB1 LOF, which was strongly biased toward upregulation of gene expression (Langer et al., 2019), and the effect we observed upon complete LOF, which was biased toward downregulation of gene expression (~240 upregulated genes and ~440 downregulated genes).

Finally, we found that SMARCB1 expression is essential for cell-cell and cell-ECM interactions in hPSCs. The effect of SMARCB1 LOF on these interactions may explain, at least in part, the abnormal self-renewal and differentiation capacity of the cells. Since these interactions were not reported to be impaired by partial SMARCB1 LOF (Langer et al., 2019), we assume that even low SMARCB1 levels are sufficient to maintain the normal cell-cell and cell-ECM interactions in hPSCs. It was shown previously that SMARCB1 plays a vital role in the maintenance of normal cell adhesion and morphology in cancer and transformed cells, such as 293, MCF7 (Caramel et al., 2008), and NCCIT (You et al., 2013) cells. In addition, Darr et al. (2014) found that re-expression of SMARCB1 in RT cells leads to upregulation of GO terms related to cell adhesion, extracellular

(F) Representative images of Phalloidin (green) and OCT4 (red) immunostaining. Upper panels: low magnification. Scale bar, 100 μm. Bottom panels: high magnification. Scale bar, 50 μm. The actin fence organization of the actin fibers appears at the edges of the control colonies (white arrows), but not in the SMARC1 LOF cells.

(G) EBs derived from control and KIKO cells upon Dox withdrawal at two different time points. Scale bar, 500 μm.

(H) A representative image of western blot for activated β-catenin (tubulin shown as a loading control).

(I) Cell number quantification of SMARCB1 LOF and control colonies grown attached to the plate upon single-cell passaging in the presence or absence of CHIR99021 treatment. n = 3; ns, not significant, $^*p < 0.05$. Statistical analysis was performed using one-way ANOVA with FDR correction.

space, and integrin pathway. These observations strongly support our results, which provide the first indication for the role of SMARCB1 in cell interactions in hPSCs. Our results further show that SMARCB1 LOF impairs the unique actin fence organization of actin fibers in hPSC colonies. This finding is also supported by previous studies in RT cell lines, which reveal the interplay between SMARCB1 and actin fibers organization (Caramel et al., 2008; Darr et al., 2015; Medjkane et al., 2004). Yet, by contrast to RT cells (Darr et al., 2015), it appears that in hPSCs SMARCB1 expression is not required for the initial adhesion to the plate. Importantly, the observation that SMARCA4 LOF leads to downregulation of genes involved in cell adhesion (Zhang et al., 2014) shows that other SWI/SNF core subunits also participate in the regulation of cell-cell and cell-ECM interactions in hPSCs. On the other hand, the fact that the morphological changes caused by SMARCA4 LOF were completely different from those seen upon SMARCB1 LOF indicates a unique role for each of these subunits in cell adhesion.

Our results suggest that, in the case of SMARCB1, the regulation of cell interactions is mediated at least in part via the WNT pathway, as SMARCB1 LOF leads to downregulation of several genes which are part of this pathway as well as of activated β-catenin. This conclusion is further supported by the partial rescue of the morphological phenotype by the WNT activator CHIR99021. Interestingly, SMARCB1 LOF during mouse limb morphogenesis, as well as in RT cell lines, results in the opposite effect, i.e., abnormal activation of the WNT pathway (Mora-Blanco et al., 2014). This dissimilar effect of SMARCB1 LOF suggests a complex and cell-dependent regulation of the WNT pathway by SMARCB1. Finally, it has been shown that a catenin-F-actin-cadherin complex is required for normal cell adhesion (Pieters and van Roy, 2014; Yonemura et al., 1995, 2010). Our observations regarding the effect of SMARCB1 LOF on cell adhesion, actin organization, and β-catenin activation suggest a possible regulation of this complex by SMARCB1 in hPSCs.

In summary, the role of the SWI/SNF complex and its SMARCB1 subunit in epigenetic regulation of diverse cell types has been extensively studied during the last years. In the context of hPSCs, it has been shown that the SWI/SNF complex binds to enhancers and super-enhancers of genes, which control the self-renewal and differentiation of the cells (Langer et al., 2019; Zhang et al., 2014). In the current study, we explored the effects of SMARCB1 complete LOF and overexpression on hPSCs. Overall, the results of our study, along with Langer et al.'s and Terada et al.'s results, show that SMARCB1 levels have to be accurately regulated in order to maintain the self-renewal capacity and pluripotency of hPSCs and that any deviation from normal SMARCB1 expression levels affects hPSC fate in a different manner. We further define, for the first time, SMARCB1 as a critical regulator of normal cell-cell and cell-ECM interactions of hPSCs.

EXPERIMENTAL PROCEDURES

Establishment of SMARCB1 Conditional Expression System

Establishment of KI cells: SMARCB1 cDNA was cloned into AAVS1-TRE3G-EGFP donor plasmid. A gRNA targeting AAVS1 locus was designed and cloned into pSpCas9(BB)-2A-GFP plasmid. The donor and the pSpCas9 plasmids were cotransfected into hPSCs. Puromycin-resistant clones were selected and subjected to PCR validation of the appropriate integration.

Establishment of KIKO cells: gRNAs targeting sequences upstream and downstream SMARCB1 exon 2 (see Figure S1) were designed and cloned into pSpCas9(BB)-2A-GFP plasmid. KI cells were cotransfected with both plasmids. Single-cell-derived clones were obtained after GFP FACS sorting and subjected to PCR analysis to detect exon 2 excision. For a detailed description, see Supplemental Experimental Procedures.

To maintain normal SMARCB1 levels during the derivation and expansion of KIKO cells, the cells were grown in the presence of low Dox concentration (12 ng/mL Dox, Sigma-Aldrich). For complete LOF experiments, the Dox was withdrawn from the KIKO cells (see Figure 1A). For gain-of-function experiments, KI cells were grown in the presence of high Dox concentration (50 ng/mL).

Statistics

All data were generated from at least three biological replicates performed independently. p values were calculated by either Student's t tests or one-way ANOVA with adjusted p value for multiple testings using GraphPad Prism software. For additional information regarding the statistical analyses, see figure legends.

For additional procedures, see Supplemental Experimental Procedures.

Data and Code Availability

The RNA-seq data have been deposited in NCBI's GEO repository. GEO: GSE158842.

SUPPLEMENTAL INFORMATION

Supplemental Information can be found online at https://doi.org/10.1016/j.stemcr.2020.10.002.

AUTHOR CONTRIBUTIONS

I.C-G. designed the study, performed the experiments, and analyzed the data. E.L. performed the experiments. L.A. designed the study, analyzed the data, and wrote the manuscript. O.Y performed the RNA-seq. H.W.B-A. performed the bioinformatics analysis. A.U. conceived and designed the study, analyzed the data, and wrote the manuscript.

ACKNOWLEDGMENTS

The authors thank Dr. Su-Chun Zhang (University of Wisconsin, Madison, WI, USA) for kindly providing the AAVS1-TRE3G-EGFP

donor plasmid. This work was funded in part by the Israel Cancer Association (ICA), grant no. 20181072.

Received: January 8, 2020
Revised: October 1, 2020
Accepted: October 2, 2020
Published: October 29, 2020

REFERENCES

Alver, B.H., Kim, K.H., Lu, P., Wang, X., Manchester, H.E., Wang, W., Haswell, J.R., Park, P.J., and Roberts, C.W.M. (2017). The SWI/SNF chromatin remodelling complex is required for maintenance of lineage specific enhancers. Nat. Commun. *8*, 14648.

Bibikova, M., Laurent, L.C., Ren, B., Loring, J.F., and Fan, J.-B. (2008). Unraveling epigenetic regulation in embryonic stem cells. Cell Stem Cell *2*, 123–134.

Birenboim, R., Markus, A., and Goldstein, R.S. (2013). Simple generation of neurons from human embryonic stem cells using agarose multiwell dishes. J. Neurosci. Methods *214*, 9–14.

Caramel, J., Quignon, F., and Delattre, O. (2008). RhoA-dependent regulation of cell migration by the tumor suppressor hSNF5/INI1. Cancer Res. *68*, 6154–6161.

Darr, J., Klochendler, A., Isaac, S., and Eden, A. (2014). Loss of IGFBP7 expression and persistent AKT activation contribute to SMARCB1/Snf5-mediated tumorigenesis. Oncogene *33*, 3024–3032.

Darr, J., Klochendler, A., Isaac, S., Geiger, T., and Eden, A. (2015). Phosphoproteomic analysis reveals Smarcb1 dependent EGFR signaling in malignant rhabdoid tumor cells. Mol. Cancer *14*, 167.

Gao, X., Tate, P., Hu, P., Tjian, R., Skarnes, W.C., and Wang, Z. (2008). ES cell pluripotency and germ-layer formation require the SWI/SNF chromatin remodeling component BAF250a. Proc. Natl. Acad. Sci. U S A *105*, 6656–6661.

Ho, L., Ronan, J.L., Wu, J., Staahl, B.T., Chen, L., Kuo, A., Lessard, J., Nesvizhskii, A.I., Ranish, J., and Crabtree, G.R. (2009). An embryonic stem cell chromatin remodeling complex, esBAF, is essential for embryonic stem cell self-renewal and pluripotency. Proc. Natl. Acad. Sci. U S A *106*, 5181–5186.

Kidder, B.L., Palmer, S., and Knott, J.G. (2009). SWI/SNF-Brg1 regulates self-renewal and occupies core pluripotency-related genes in embryonic stem cells. Stem Cells *27*, 317–328.

Kim, J.K., Huh, S.O., Choi, H., Lee, K.S., Shin, D., Lee, C., Nam, J.S., Kim, H., Chung, H., Lee, H.W., et al. (2001). Srg3, a mouse homolog of yeast SWI3, is essential for early embryogenesis and involved in brain development. Mol. Cell. Biol. *21*, 7787–7795.

Klochendler-Yeivin, A., Fiette, L., Barra, J., Muchardt, C., Babinet, C., and Yaniv, M. (2000). The murine SNF5/INI1 chromatin remodeling factor is essential for embryonic development and tumor suppression. EMBO Rep. *1*, 500–506.

Langer, L.F., Ward, J.M., and Archer, T.K. (2019). Tumor suppressor SMARCB1 suppresses super-enhancers to govern hESC lineage determination. Elife *8*, e45672.

Lee, R.S., Stewart, C., Carter, S.L., Ambrogio, L., Cibulskis, K., Sougnez, C., Lawrence, M.S., Auclair, D., Mora, J., Golub, T.R., et al. (2012). A remarkably simple genome underlies highly malignant pediatric rhabdoid cancers. J. Clin. Invest. *122*, 2983–2988.

Masliah-Planchon, J., Bièche, I., Guinebretière, J.-M., Bourdeaut, F., and Delattre, O. (2014). SWI/SNF chromatin remodeling and human malignancies. Annu. Rev. Pathol. Mech. Dis. *10*, 145–171.

Medjkane, S., Novikov, E., Versteege, I., and Delattre, O. (2004). The tumor suppressor hSNF5/INI1 modulates cell growth and actin cytoskeleton organization. Cancer Res. *64*, 3406–3413.

Meshorer, E., and Misteli, T. (2006). Chromatin in pluripotent embryonic stem cells and differentiation. Nat. Rev. Mol. Cell Biol. *7*, 540–546.

Mora-Blanco, E.L., Mishina, Y., Tillman, E.J., Cho, Y.J., Thom, C.S., Pomeroy, S.L., Shao, W., and Roberts, C.W.M. (2014). Activation of β-catenin/TCF targets following loss of the tumor suppressor SNF5. Oncogene *33*, 933–938.

Nakayama, R.T., Pulice, J.L., Valencia, A.M., McBride, M.J., McKenzie, Z.M., Gillespie, M.A., Ku, W.L., Teng, M., Cui, K., Williams, R.T., et al. (2017). SMARCB1 is required for widespread BAF complex-mediated activation of enhancers and bivalent promoters. Nat. Genet. *49*, 1613–1623.

Närvä, E., Stubb, A., Guzmán, C., Blomqvist, M., Balboa, D., Lerche, M., Saari, M., Otonkoski, T., and Ivaska, J. (2017). A strong contractile actin fence and large adhesions direct human pluripotent colony morphology and adhesion. Stem Cell Reports *9*, 67–76.

Nichols, J., and Smith, A. (2009). Naive and primed pluripotent states. Cell Stem Cell *4*, 487–492.

Pieters, T., and van Roy, F. (2014). Role of cell-cell adhesion complexes in embryonic stem cell biology. J. Cell Sci. *127*, 2603–2613.

Schaniel, C., Ang, Y.-S., Ratnakumar, K., Cormier, C., James, T., Bernstein, E., Lemischka, I.R., and Paddison, P.J. (2009). Smarcc1/Baf155 couples self-renewal gene repression with changes in chromatin structure in mouse embryonic stem cells. Stem Cells *27*, 2979–2991.

Stubb, A., Guzmán, C., Närvä, E., Aaron, J., Chew, T.-L., Saari, M., Miihkinen, M., Jacquemet, G., and Ivaska, J. (2019). Superresolution architecture of cornerstone focal adhesions in human pluripotent stem cells. Nat. Commun. *10*, 4756.

Terada, Y., Jo, N., Arakawa, Y., Sakakura, M., Yamada, Y., Ukai, T., Kabata, M., Mitsunaga, K., Mineharu, Y., Ohta, S., et al. (2019). Human pluripotent stem cell-derived tumor model uncovers the embryonic stem cell signature as a key driver in atypical teratoid/rhabdoid tumor. Cell Rep. *26*, 2608–2621.

Wang, X., Lee, R.S., Alver, B.H., Haswell, J.R., Wang, S., Mieczkowski, J., Drier, Y., Gillespie, S.M., Archer, T.C., Wu, J.N., et al. (2017). SMARCB1-mediated SWI/SNF complex function is essential for enhancer regulation. Nat. Genet. *49*, 289–295.

Yan, Z., Wang, Z., Sharova, L., Sharov, A.A., Ling, C., Piao, Y., Aiba, K., Matoba, R., Wang, W., and Ko, M.S.H. (2008). BAF250B-associated SWI/SNF chromatin-remodeling complex is required to maintain undifferentiated mouse embryonic stem cells. Stem Cells *26*, 1155–1165.

Yilmaz, A., and Benvenisty, N. (2019). Defining human pluripotency. Cell Stem Cell *25*, 9–22.

Yonemura, S., Itoh, M., Nagafuchi, A., Tsukita, S., Konstantopoulos, K., and Wirtz, D. (1995). Cell-to-cell adherens junction

formation and actin filament organization: similarities and differences between non-polarized fibroblasts and polarized epithelial cells. J. Cell Sci. *108* (Pt 1), 127–142.

Yonemura, S., Wada, Y., Watanabe, T., Nagafuchi, A., and Shibata, M. (2010). alpha-Catenin as a tension transducer that induces adherens junction development. Nat. Cell Biol. *12*, 533–542.

You, J.S., de Carvalho, D.D., Dai, C., Liu, M., Pandiyan, K., Zhou, X.J., Liang, G., and Jones, P.A. (2013). SNF5 is an essential executor of epigenetic regulation during differentiation. PLoS Genet. *9*, 1–14.

Zhang, X., Li, B., Li, W., Ma, L., Zheng, D., Li, L., Yang, W., Chu, M., Chen, W., Mailman, R.B., et al. (2014). Transcriptional repression by the BRG1-SWI/SNF complex affects the pluripotency of human embryonic stem cells. Stem Cell Reports *3*, 460–474.

Stem Cell Reports, Volume *15*

Supplemental Information

Human Pluripotent Stem Cell Fate Regulation by SMARCB1

Ilana Carmel-Gross, Etgar Levy, Leah Armon, Orly Yaron, Hiba Waldman Ben-Asher, and Achia Urbach

Supplemental Information

Supplemental Figures

Figure S1

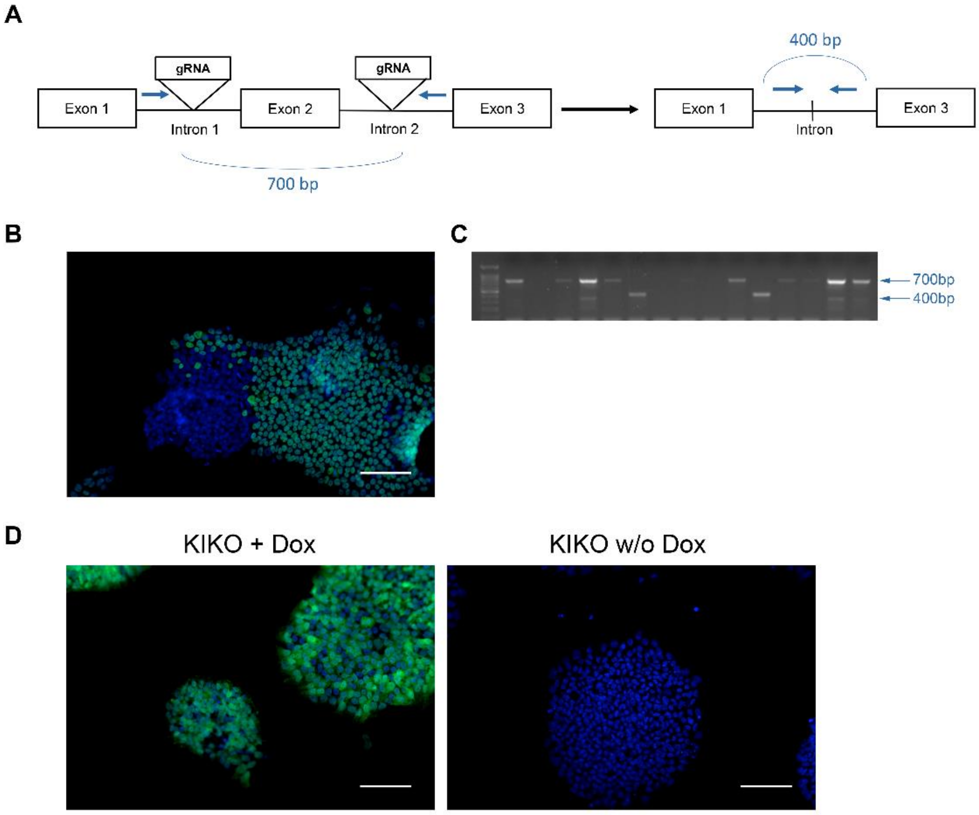

Figure S1 – Targeting *SMARCB1* by the CRISPR/Cas9 system. A. The combination of two gRNAs directed upstream and downstream to exon 2 leads to the excision of the entire exon. The blue arrows represent PCR primers flanking the sequences targeted by the gRNAs. The removal of the exon, results in a decrease in the PCR product size from 700 bp to 400 bp. **B.** SMARCB1 immunostaining of hPSC population following introduction of the CRISPR system. Note the absence of SMARCB1 from some of the cells. Green – SMARCB1. Blue – DAPI. **C.** Representative PCR results (using the abovementioned primers) showing WT, heterozygous and homozygous hPSC clones. **D.** Immunostaining of single cell derived KIKO clones from hiPSCs in the presence of Dox or 96h following Dox withdrawal. Green – SMARCB1. Blue – DAPI. Scale bar: 100μm.

Figure S2

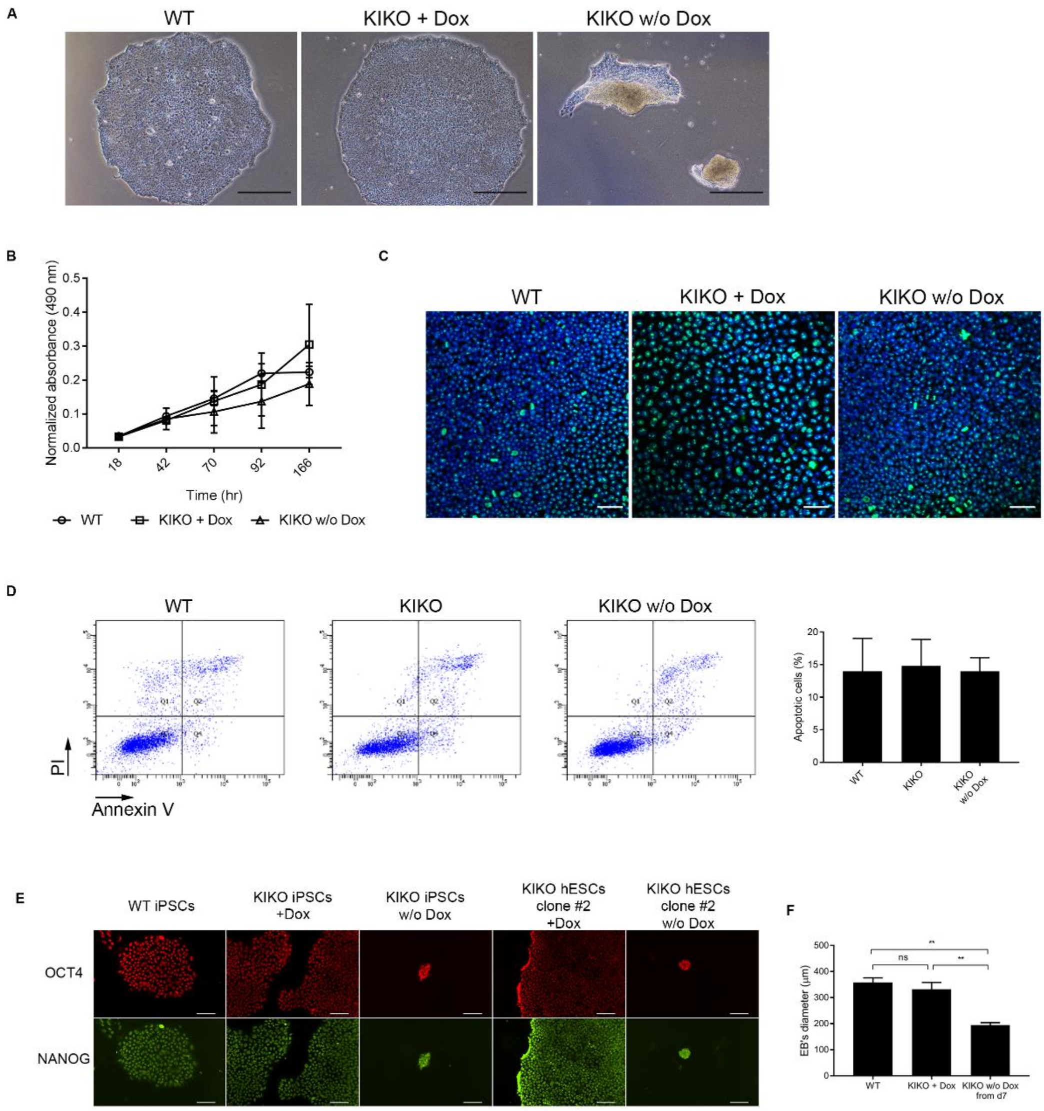

Figure S2 – SMARCB1 complete LOF effect on hPSC fate. A. Phase-contrast images of representative SMARCB1 LOF and control hiPSC colonies. **B.** MTS assay for cell viability and proliferation for SMARCB1 LOF and control hESCs. No statistical significant differences were found at any of the time points. Statistical analysis was done by two-way ANOVA, n=3. **C.** Ki67 immunostaining of SMARCB1 LOF and control hESCs. Green - Ki67, Blue – DAPI. Scale bar: 100 µm **D.** Apoptosis assay based on AnnexinV. Representative FACS results for each sample and quantification of three biological repeats are presented. No statistical significant difference was found according to one-way ANOVA. **E.** Representative Oct4 and Nanog immunostaining results for KIKO iPSCs and an additional clone of KIKO hESCs upon single cell passaging. Scale bar: **F.** Size measurement of EBs derived from control or SMARCB1 LOF cells. Dox withdrawal from d0 resulted in very few EBs, therefore size measurement was not applicable for these cells. One-way ANOVA with Tukey post-test. P<0.001.

Supplemental Tables

Table S1 – primers used for qRT-PCR

Primer name	Forward	Reverse
MIXL1	GGCGTCAGAGTGGGAAATCC	GCAGTTCACATCTACCTCAAGAG
PAX6	TCTTTGCTTGGGAAATCC	CTGCCCGTTCAACATCCTTAG
CDX2	GGCAGCCAAGTGAAAACCAG	GGTGATGTAGCGACTGTAGTGAA
EOMES	CCCACTACAATGTGTTCGTAGAG	CATTTTGTTGCCCTGCATGTT
FOXA2	GGTGTCTGAGGAGTCGGAGA	CCTCGGGCTCTGCATAGTAG
SOX17	AAGGGCGAGTCCCGTATC	TTGTAGTTGGGGTGGTCCTG
NEUROG2	CGTCCTCCTCCGTGTCCTCCAATTCCACCT	AAGAGAAAGGGGAGGAGCGTCAGTCCGCTC
NR4A2	AGTCTGATCAGTGCCCTCGT	TATGCTGGGTGTCATCTCCA
T	TCAGCAAAGTCAAGCTCACCA	CCCCAACTCTCACTATGTGGATT
GATA6	AGAAACGCCGAGGGTGAAC	AGTTGGAGTCATGGGAATGGAA
GDNF	CTGCCTGGTGCTGCTCCACA	AGCTGCAGCCTGCCGATTCC
NCAM1	GATGCGACCATCCACCTCAA	TCTCCGGAGGCTTCACAGGTA
βACTIN	CACCTTCTACAATGAGCTGCGTGTG	ATAGCACAGCCTGGATAGCAACGTAC

Supplemental Experimental Procedures

Establishment of SMARCB1 conditional expression system

Establishment of KI cells: *SMARCB1* cDNA was cloned into AAVS1-TRE3G-EGFP donor plasmid. kindly donated by Dr. Su-Chun Zhang (Qian et al., 2014) using Mlul and Sall restriction enzymes to replace the EGFP sequence. gRNA (GTCCCCTCCACCCCACAGTG) targeting AAVS1 locus was designed and cloned into pSpCas9(BB)-2A-GFP plasmid (Addgene #48138) as described in (Ran et al., 2013). The donor and the pSpCas9 plasmids were cotransfected into hPSCs using LipofectaminStem (Thermo). Puromycin resistant clones (indicating donor sequence integration into an active gene, most probably *AAVS1* locus upon homologous recombination) were selected and subjected to PCR validation of the appropriate integration, using the following primers: set 1: forward: CAAACAGCATAAGCTGGTCAC reverse: CTGACGCTCTTGACGATTTTGAC set 2: forward: GAAGAGTTCTTGCAGCTCG reverse: CTTTGAGCTCTACTGGCTTC.

Establishment of KIKO cells: gRNAs targeting sequences upstream (CTGCCGAAAGCGTGGCGCCT) and downstream (AGCCTTGGCCTTAGTCGGGC) SMARCB1 exon 2 (see **Figure S1**) were designed and cloned into pSpCas9(BB)-2A-GFP plasmid (addgene #48138) as described in (Ran et al., 2013). KI cells were cotransfected with both plasmids. Single cell derived-clones were obtained following GFP FACS sorting and subjected to PCR analysis to detect exon 2 excision using the following primers: forward primer: CCTTTCGGAACTTGGTTCTGTTG. reverse primer: GATGTGCTCCAGGAAGCAAG as described in Figure S1.

Cell culture

hESCs (HUES13) and hiPSCs were grown feeder-free on vitronectin (PeproTech) coated plates in mTeSR1 medium (STEMCELL Technologies). Cells were passaged either with ReLeSR (STEMCELL Technologies) or accutase (Sigma) for single cell passaging. The cells were treated with 10uM ROCK inhibitor (Y27632, PeproTech) overnight upon single cell passaging.

Single cell passaging assay

Dox was withdrawal for 72h prior to the assay. Cells were harvested as single cells and plated at 100,000cells/well of 12 well plate. The morphology and cell number were evaluated 96h post the single cell seeding. For WNT pathway experiment CHIR99021 (2μM, Tocris) was added starting from the day of Dox withdrawal.

Reprogramming of somatic cells into hiPSCs.

Fibroblasts obtained from hPSC (CSES7 line) derived teratoma, were reprogrammed into hiPSCs using the CoMIP plasmid (Addgene #63727) according to the protocol published by (Diecke et al., 2015). CSES7 hPSCs were kindly provided by Prof. Benvenisty, The Hebrew University, Israel.

Western Blot assay

Western blot was performed according to the usual procedures. The following antibodies were used: SMARCB1 (Abcam, ab58209, 1:500), OCT4 (Santa Cruz Biotechnologies, sc-5279, 1:500), Nanog (Abcam, ab80892, 1:1000 or R&D Systems, AF1997, 1:200), activated β-catenin (Millipore, 05-665, 1:1000), α-Tubulin (Cell Signaling, 2144, 1:1000) and GAPDH (Millipore, ABS16, 1:1000).

Immunoflurescence

Cells were fixed with 4% paraformaldehyde and permeabilized for 5 min with 0.1% Triton in PBS. Blocking was performed with 5% goat serum, 0.05% Triton X100 in PBS. Primary antibodies were applied for two hours in RT, washed and incubated with fluorophore conjugated secondary antibodies for one hour at RT. Nuclei were stained with DAPI. The following primary antibodies were SMARCB1 (Abcam, ab192864, 1:500), NF-H (Sigma, N4142, 1:250), Oct4 (Santa Cruz Biotechnologies, sc-5279, 1:500), NANOG (R&D systems, AF1997, 1:100), and Ki67 (Invitrogen, 14-5698-82, 1:500). Actin fibers were stained with Phalloidin-iFluor488 (Abcam, 1:1000).

qRT-PCR

Total RNA was extracted using a RiboEx Total RNA purification solution (GeneAll), followed by reverse transcription using iScript cDNA Synthesis Kit (BioRad). qRT-PCR was performed using FastStart Universal SYBR Green Master Mix (Roche). β-actin was used for normalization. For primers list see **Table S1.**

Aggregation (EBs formation) assay

To generate uniform size cell aggregates the cells were seeded in agarose microwells as follows: Agarose and NaCl solution was poured into 256 MicroTissues 3D Petri Dish micro-mold (Sigma-Aldrich, Z764000) to prepare microwells as described in (Birenboim et al. 2013). Cells were harvested with Accutase (Sigma-Aldrich) and plated into the 256 microwells (750,000 cells per microwell plate, ~3000 cells per each well). The cells were grown in EBs medium - DMEM/F12 (Biological Industries), 10% KOSR (Thermo Fisher Scientific), 1% MEM non-essential amino acids (Biological Industries), 0.1 mM β-mercaptoethanol, 1 mM glutamine (Biological Industries) and 1% Pen/Strep (Biological Industries). EBs grown in molds for two weeks and then transferred to a non-adherent plate and analyzed EB formation at day 30.

Neuronal differentiation

Neuronal differentiation was performed according to (Birenboim et al. 2013) with minor modifications. Cells ($7.5X10^5$) were resuspended in 100μl EBs medium supplemented with 10μM SB431542 (Tocris), 2μM dorsomorphin dihydrochloride (Tocris) and Y27632 (PeproTech). Next, the cells were transferred to microwells (see above). Four days after the initial seeding the medium was changed to EBs medium. At day 14 cells aggregates were transferred to פoly-L-Lysine (Sigma-Aldrich) and laminin (Sigma-Aldrich) coated plate in neural induction medium (DMEM/F12, 1mM L-Glutamin, 1% Pen/Strep, 2% B27 (Thermo Fisher Scientific), 10ng/ml NGF (Alomone Labs), 10ng/ml NT-3 (Alomone Labs), 10ng/ml BDNF (Alomone Labs) and 10ng/ml GDNF (PeproTech).

Endodermal and ectodermal differentiation

STEMDiff Trilineage Differentiation Kit (Stemcell Technologies) was used for endodermal and ectodermal differentiation according to manufacturer's instructions. For SMARCB1 LOF, Dox was removed from KIKO cells one day before seeding for endoderm differentiation and one day after seeding for ectodermal differentiation. Overall the KIKO cells were without Dox for seven days at the end of each protocol or with Dox for the entire period as control.

Mesodermal differentiation

Cells were seeded at low density ($9X10^4$ cells/12well) two days prior to mesodermal differentiation induction. Then, the cells were cultured for 2 days in RPMI1640 medium (Biological Industries) supplemented with B27 without insulin (Thermo Fisher Scientific) and 8 μM CHIR99021 (Tocris). Next, the medium was changed to RPMI1640 supplemented with B27 without insulin alone, for two additional days. For SMARCB1 LOF experiment Dox was removed from KIKO cells one day before cell seeding.

Teratoma formation

Cells (2X 10cm plates 90% confluent) were resuspended in 100μl mTeSR and mixed with 100μl Matrigel (Corning). Then the cells were subcutaneously injected into SCID mice (NOD.CB17-Prkdc-scid, Envigo). Teratomas were harvested 8 weeks following the transplantation, fixed with 4% formaldehyde and embedded in paraffin. The teratomas were stained by standard H&E staining protocols. All animal procedures were conducted according to animal care guidelines approved by the Institutional Animal Care and Use Committee at Bar Ilan University (approval #11-02-2018).

Apoptosis assay

Apoptosis analysis was performed using MEBCYTO-Apoptosis Kit (MBL) according to manufacturer's instructions for FACS analysis. For SMARCB1 LOF Dox was removed seven days prior to the analysis.

Proliferation assay

Proliferation assay was using the CellTiter 96® AQueous One Solution Cell Proliferation Assay (MTS) (Promega). The starting seeding density was 1000 cells/well (96 well plate). For SMARCB1 LOF experiment Dox removed from KIKO cells at seeding day.

ECM adhesion assay

For cellular adhesion assay we used the CytoSelect™ 48-Wll Cell Adhesion Assay (ECM Array, Colorimetric, Cell Biolabs), According to the manufacturer's instructions. Dox was withdrawn 96h prior to the assay. Cells were harvested as single cells. 80,000 cells/well were plated. The adhesion was evaluated 90min after cell seeding. The vitronectin experiment was done separately by coating regular 48 well plate with vitronectin following the same analysis as for the other ECM proteins.

RNAseq

RNA quality control - For QC of purified RNA, absorbance ratios A260:A280 and A260:A230 were assessed with NanoDrop 2000. The integrity of RNA was evaluated based on RIN acquired via capillary gel electrophoresis performed using Agilent 4200 TapeStation in combination with Agilent RNA ScreenTape System (Agilent Technologies). All RNA samples went through DNase Treatment Kit (Qiagen) before proceeding to the next step.

PolyA selection and library preparation - For library preparation NEBNext RNA ultra II RNA library preparation kit (NEB) was used. All RNA samples underwent PolyA selection following the manufacturers' protocols. Samples were multiplexed using suitable molecular barcodes and resulting cDNA pools were processed according to the NextSeq System Denature and Dilute Libraries guide. (Illumina). Quantification and quality control of the libraries were done using Qubit fluorimeter and Agilent 4200 TapeStation.

Next-generation sequencing - Single-read sequencing of the libraries with a read length of 75 was performed with NextSeq 500 Sequencing System using NextSeq 500/550 High Output v2 kit (75 cycles) (20024906 Illumina). PhiX Control v3 (Illumina) was added at 1% to all pools as an internal control before the sequencing.

Bioinformatics analysis – Sequenced reads were mapped to the human reference genome sequences (hg19) using STAR. The aligned reads were quantitated by Htseq. The normalization and differentially expressed genes test were implemented by DESeq2. An arbitrary cutoff of at least 2-fold and p-value adjusted for multiple testing < 0.05 were chosen to define a differentially expressed gene. Geneanalytics tool was used for enrichment analysis on the differentially expressed genes between each group.

The raw data have been deposited in the NCBI Sequence Read Archive (SRA) with SRA accession number: PRJNA574055.

References

Diecke, S., Lu, J., Lee, J., Termglinchan, V., Kooreman, N.G., Burridge, P.W., Ebert, A.D., Churko, J.M., Sharma, A., Kay, M.A., et al. (2015). Novel codon-optimized mini-intronic plasmid for efficient, inexpensive and xeno-free induction of pluripotency. Sci. Rep. *5*, 8081.

Qian, K., Huang, C.-L., Chen, H., Blackbourn, L.W., Chen, Y., Cao, J., Yao, L., Sauvey, C., Du, Z., Zhang, S.-C., et al. (2014). A Simple and Efficient System for Regulating Gene Expression in Human Pluripotent Stem Cells and Derivatives. Stem Cells *32*, 1230–1238.

Ran, F.A., Hsu, P.D., Wright, J., Agarwala, V., Scott, D.A., and Zhang, F. (2013). Genome engineering using the CRISPR-Cas9 system. Nat. Protoc. *8*, 2281–2308.
